# An Understandable, Extensible, and Reusable Implementation of the Hodgkin-Huxley Equations Using Modelica

**DOI:** 10.3389/fphys.2020.583203

**Published:** 2020-10-02

**Authors:** Christopher Schölzel, Valeria Blesius, Gernot Ernst, Andreas Dominik

**Affiliations:** ^1^Life Science Informatics, Technische Hochschule Mittelhessen - University of Applied Sciences, Gießen, Germany; ^2^Vestre Viken Hospital Trust, Kongsberg, Norway; ^3^Psychological Institute, University of Oslo, Oslo, Norway

**Keywords:** understandability, cognitive load theory, Modelica, mathematical modeling, software engineering, model engineering, Hodgkin-Huxley, action potential

## Abstract

The Hodgkin-Huxley model of the squid giant axon has been used for decades as the basis of many action potential models. These models are usually communicated using just a list of equations or a circuit diagram, which makes them unnecessarily complicated both for novices and for experts. We present a modular version of the Hodgkin-Huxley model that is more understandable than the usual monolithic implementations and that can be easily reused and extended. Our model is written in Modelica using software engineering concepts, such as object orientation and inheritance. It retains the electrical analogy, but names and explains individual components in biological terms. We use cognitive load theory to measure understandability as the amount of items that have to be kept in working memory simultaneously. The model is broken down into small self-contained components in human-readable code with extensive documentation. Additionally, it features a hybrid diagram that uses biological symbols in an electrical circuit and that is directly tied to the model code. The new model design avoids many redundancies and reduces the cognitive load associated with understanding the model by a factor of 6. Extensions can be easily applied due to an unifying interface and inheritance from shared base classes. The model can be used in an educational context as a more approachable introduction to mathematical modeling in electrophysiology. Additionally the modeling approach and the base components can be used to make complex Hodgkin-Huxley-type models more understandable and reusable.

## 1. Introduction

Since 1952, when Alan Hodgkin and Andrew Huxley published their conductance-based model of the action potential generation in the squid giant axon, the Hodgkin-Huxley (HH) model has been the basis of countless research projects to further the understanding of ionic currents and action potentials in neurons and cardiac myocytes (Hodgkin and Huxley, 1952). Today's models feature more types of ion channels and pumps than the three channels identified by Hodgkin and Huxley, but they still use the same electrical analogy and equation structure, which is why we will call them HH-type models in the following (Courtemanche et al., 1998; Inada et al., 2009; Fabbri et al., 2017; Bai et al., 2018). Although other types of ion channel models, such as Markov Models, are emerging, HH-type models are still the gold standard (Winslow et al., 1999; Fink and Noble, 2009). The descriptions of these HH-type models usually follow one of three explanatory approaches: Either the differential equations are given directly with a short biological explanation of the major variables or a diagram of the electrical analogy is shown and explained in biological terms or a combination of both. Often a biological drawing of the cell is also provided, but it is only used to explain the modeled concepts and not tied to the model equations themselves. This holds for research articles (Courtemanche et al., 1998; Inada et al., 2009), simulation toolkits (Hines and Carnevale, 1997; Jordan et al., 2020) and textbooks (Voit, 2013; Gerstner et al., 2014). However, these approaches pose significant challenges for novices and limit the productivity of experts: A novice has to become familiar with the formalism of differential equations or with circuit diagrams at the same time as they try to understand the model itself. Experts will have overcome this barrier already, but they are also faced with much more complex HH-type models that can easily grow to over 100 equations. These equations all interact with each other in a multitude of feedback loops, making it extremely difficult to spot small errors or to reproduce and extend the model. The risk associated with these barriers is 2-fold: On the one hand, students may choose not to specialize in systems biology or electrophysiology, because they perceive the field to be too difficult. On the other hand, a published model that is described in this way may generate new insights, but prove to be too hard to reuse and extend. For example, the latter seems to be the case for a model by Inada et al. (2009) (116 equations) which has been labeled as “groundbreaking” (Noble et al., 2012) but has only been used for simulations by two other research groups in 10 years. It becomes apparent that there is room for improving the understandability of HH-type models and that this should be a goal of both the initial model design and its presentation in scientific and instructional material.

One area from which such an improvement may originate is software engineering, because software development faces similar problems of understandability: The building blocks of source code are easy to grasp, but creating and maintaining projects with millions of lines of code requires additional organization. A widely established solution to handle this complexity is modularization. Instead of overseeing the whole project at once, software engineers identify individual functions and parts of a system and create small modules to represent them. Each component only has a few lines of code and a limited number of connections to the outside world which makes it understandable. To form the whole system, the modules can be connected at a higher level of abstraction, where each of them can be considered to be a single entity.

In recent years, multiple researchers have advocated to borrow concepts from software engineering for systems biology, culminating in the formulation of the term “model engineering” (Hellerstein et al., 2019). In accordance with this movement, we found that the modeling language and consistent application of relevant language characteristics can have a significant impact on the model quality (Schölzel et al., 2020). In this paper we therefore present a novel modular implementation of the original HH model that is based on the electrical analogy, but explains and visualizes each component in biological terms. The model is written in the modeling language Modelica and makes heavy use of the features of this language and of software engineering techniques.

Due to the aforementioned anticipated benefits of these techniques we pose the following research questions:

RQ1 Can the understandability of the HH model be improved by a modular implementation that bridges the gap between biological meaning and electrical analogy?
RQ2 Can a modular implementation of the HH model serve as a unifying basis for extensions and therefore facilitate the creation of more complex HH-type modules?

For the investigation of these questions, the term *understandability* is central, as our model does not differ from other solutions in terms of its output, but only its presentation. We may find our assessment of what makes a model more or less understandable intuitive, but in a scientific context it is not sufficient to rely on intuition alone. This is especially true, when it involves reasoning about the experiences of other people which may have quite a different background and perspective. Therefore, a model for understandability is needed that is based on scientific evidence. For this task we use cognitive load theory (CLT), a popular and well-validated theory in cognitive psychology which frames understandability in terms of the amount of items that have to be kept in active working memory and the degree of interactivity between them.

CLT is introduced in more detail in section 2.1 along with model engineering, the language Modelica, and the biological basis of the Hodgkin-Huxley model followed by an overview over the software engineering concepts that we apply and the resulting model structure. Section 2 describes our rationale for reducing cognitive load, for the component hierarchy, and the design of the individual model components. Section 3 then shows and explains the resulting model code including the graphical representation followed by a discussion of the implications of the new model structure for understandability and extensibility. Finally, section 4 sums up the answers to our research questions and discusses possible alternative approaches, limitations and future work.

## 2. Materials and Methods

### 2.1. Background

#### 2.1.1. Model Engineering

To this day, many models are still built for a single purpose without guidelines regarding code quality. However, when models grow beyond a certain point, the modeling process becomes an engineering task and the goal should not only be to produce a model that is working and mathematically sound, but also to build it with an architecture that facilitates the anticipated use cases and makes the code maintainable and accessible to other researchers (Hellerstein et al., 2019). This includes documentation, testing, naming of variables, and the use of established design patterns. In a previous work we have found the consistent use of an appropriate modeling language that is modular, descriptive, (human)-readable, open, graphical, and hybrid (MoDROGH) to be a major driving factor for model quality in terms of understandability and reproducibility (Schölzel et al., 2020).

#### 2.1.2. Modelica and Object-Oriented Programming

There are a few established and emerging languages that exhibit MoDROGH-characteristics, such as the systems biology markup language (SBML) (Hucka et al., 2003), CellML (Cuellar et al., 2003), Simscape (The MathWorks, Inc., 2020), or embedded DSLs written in Python or Julia (Olivier et al., 2005; Lopez et al., 2013; Elmqvist et al., 2016; Rackauckas et al., 2020)^1^. In our opinion, the modeling language Modelica (Mattsson and Elmqvist, 1997) is a particularly interesting example, because it is an industrial standard that emphasizes the engineering aspect of model design. In contrast to SBML, CellML, as well as Python- and Julia-based DSLs, Modelica supports full object oriented design (e.g., through model inheritance), discrete variables for the seamless integration of continuous and discrete model parts, the graphical composition of models via drag and drop, implicit differential/algebraic equations for acausal connections between components via conservation laws, cross-language export and import via the Functional Mockup Interface (Blochwitz et al., 2012), grouping of interface variables to connectors, and unrestricted mixing of implicit and explicit equation formats. It shares these features with Simscape, but unlike Simscape, Modelica provides an open environment, more flexible mechanisms for model inheritance including multiple inheritance and overwriting of variables and equations, and extensible annotations, which could, e.g., be used to implement support for ontological terms to the language. Weak points of Modelica are the lack of existing support for biological terminology and ontologies and the fact that, while the OpenModelica integrated development environment (IDE) (Fritzson et al., 2005) is open-source, many users prefer the proprietary IDE Dymola (Dassault Systèmes, 2020), which has its own compiler that is not always fully compatible with OpenModelica.

In Modelica, modularity is realized by the principles of object-oriented programming (Gamma et al., 1994). Code is structured in classes that can be instantiated to reuse the same code at different positions in a project and that can inherit behavior and interfaces from abstract base classes. This reuse is not only encouraged from one project to the following but also within one project. This corresponds with one of the guiding principles in software engineering called “don't repeat yourself” (DRY) suggesting that one should avoid writing duplicated code with only very small differences, such as constant values or variable names (Hunt and Thomas, 2000). Both “don't repeat yourself” (DRY) and object orientation are most effective, when the implemented system can be broken down into structurally similar components, which is the case for the HH model.

#### 2.1.3. The Biological Basis of the Hodgkin-Huxley Model

The Hodgkin-Huxley model explains the time course of the membrane potential of the squid giant axon during an action potential by means of three ion channels: A sodium channel lets Na^+^ cations enter the cell, which increases the potential. A delayed-rectifier potassium channel permits K^+^ cations to leave the cell, lowering the potential back toward the resting state. Finally, a leak channel is responsible for maintaining the resting potential while the other channels are closed. Both the sodium and the potassium channel have voltage-dependent gates—molecules that change their conformation with the membrane potential to activate or inactivate the channel. The sodium channel has both a fast activation gate and a slightly slower inactivation gate, allowing the channel to open for a short period of only a few milliseconds. The delayed-rectifier potassium channel only has an activation gate with slower kinetics while the leak channel is assumed to be always active.

#### 2.1.4. Cognitive Load Theory

As mentioned in the introduction, we use cognitive load theory (CLT) as a model for understandability (Sweller, 2019). In short, CLT is based on the architecture of the human brain, which has a very limited capacity for new information in the working memory, but can easily transfer stored information from long-term memory to working memory. The amount of items that have to be kept in working memory to process an information is called the cognitive load. The main driving factor of this metric is element interactivity. Independent elements can be processed one by one, but when elements have high interactivity they have to be kept in working memory simultaneously. Hence, cognitive load can be reduced in two ways: First, expertise can allow a person that has understood a concept to further on process it as a single item instead of the several items it comprises. Second, a part of cognitive load does not originate from the complexity of the taught concept itself, but from the way it is presented and can therefore be reduced by choosing appropriate methods to present the information and instruct the learner.

### 2.2. Model Design and Structure

For the sake of simplicity, we will consider the amount of variables, parameters, and equations that constitute a model or model component as an indicator of its cognitive load and thus its understandability. Our goal is therefore to produce small model components that have a low amount of elements and to reduce element interactivity by introducing clearly defined interfaces between these components that allow the learner to view one component as a single item to be kept in working memory once they understand it.

For this task we identify the following components in the HH model: The lipid bilayer that separates the ionic concentrations and therefore electrical charge on the outside and the inside of the cell; the sodium, potassium, and leak channels; the voltage-dependent gates inside the sodium and potassium channels; and the current clamp that holds the current constant in order to measure the voltage with reference to a ground electrode. All of these components reside on the same hierarchical layer, except for the gates which are components of an ion channel class.

To build the full model, two kinds of connections between non-gate components are required: First, each component has a positive electrical pin on the extracellular side and a negative electrical pin on the intracellular side, allowing current to flow through the component as positive outward current. Second, the voltage-dependent gating molecules of the sodium and potassium channels react more quickly at higher temperatures, which establishes the need for an input-output temperature connection from the lipid bilayer to the ion channels.

In the design of the Modelica model we follow our guidelines established in Schölzel et al. (2020), i.e., implementing small self-contained modules; describing only the “what” of the model, not the “how”; keeping the code human-readable with speaking names and additional documentation strings for variables and parameters; using only open-source tools (namely OpenModelica) for ease of reproduction; and adding a graphical representation for each component. We also published the full code of the model on GitHub as well as in the Supplementary Data Sheet 1 including every information required to reproduce our results. As mentioned in section 2.1.2, graphical annotations are part of the Modelica code. This encompasses connection annotations that define the coordinates of the line connecting two components and more complex icon annotations that define a component icon as vector graphic. As the latter can be quite large and tend to clutter the otherwise human-readable code, we define all icons in separate classes and include them via inheritance with the extends statement.

The model was implemented using OpenModelica version 1.16.0 (Fritzson et al., 2005) and Mo|E version 0.6.3 (Justus et al., 2017) as well as Inkscape version 0.91 (Inkscape Developers, 2020) to add the component icons. Simulations can be replicated with OpenModelica on Windows, Linux, and macOS. The code is available on GitHub under the MIT license at https://github.com/CSchoel/hh-modelica.

## 3. Results

### 3.1. Model Code

The first thing to consider in a software engineering task are the required interfaces. Hence, we start our implementation of the HH model by defining the following basic connectors:


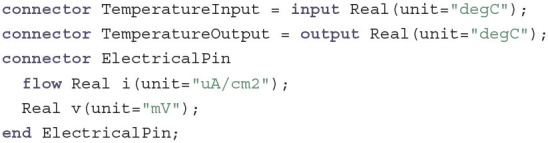


Here, TemperatureInput and TemperatureOutput follow a simple input-output relationship. All components that have a TemperatureInput will be connected to a single component with a TemperatureOutput that determines the global temperature value. ElectricalPins that are connected to each other will all have the same voltage v, but can have different currents i (indicated by the keyword flow). During compilation, Modelica will generate an equation following Kirchhoff's current law that ensures that the sum of all connected currents equals zero. This allows to connect an arbitrary number of components without having to determine the direction of the flow. For the sake of terminology and for a visual distinction PositivePin and NegativePin are introduced as subclasses without any functional difference from the base class.


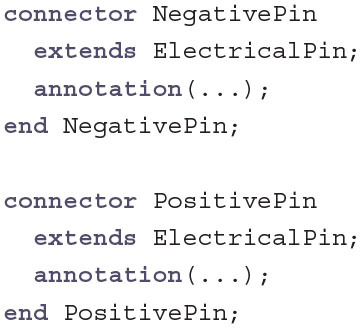


**Figure 1:**
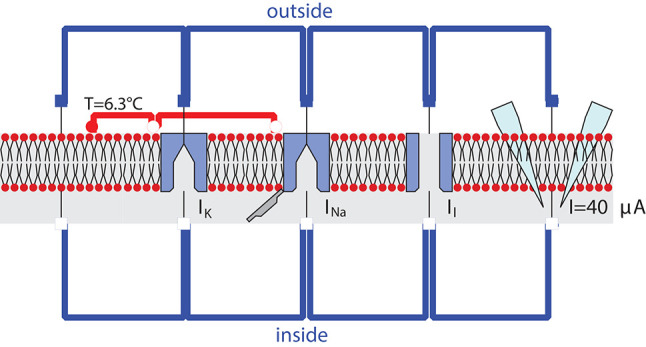
Diagram view of the modular Modelica implementation of the Hodgkin-Huxley model. Each component has a positive electrical pin on the top/outside and a negative electrical pin on the bottom/inside. From left to right the components are: LipidBilayer, PotassiumChannel, SodiumChannel, LeakChannel, and CurrentClamp. Extra connections in red represent temperature dependence of gating variables.

Annotation code that defines the connector icons is not given here for the sake of brevity and will be completely omitted for further code examples. The same is true for most of the documentation strings. These details can be viewed on GitHub and the resulting visual design can be seen in Figure 1.

Since most components will have an electrical connection both to the inside and the outside of the cell, it is beneficial to introduce another base class for those two-pin components:


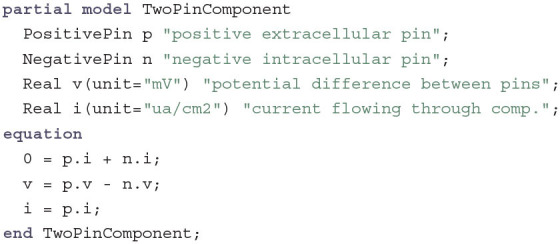


This base class already introduces three small equations that connect the positive (extracellular) and negative (intracellular) pins. The first equation again follows Kirchhoff's current law to ensure that the sum of all currents entering and leaving the components is zero. The other equations just introduce two helper variables: The variable v can be used to measure (or define) the voltage at this component as a difference between the potential at the positive and the negative pin. The variable i measures or defines the current flowing through the component from the negative to the positive pin. The model is declared as partial since the number of equations and variables is not balanced. It does not yet specify the current-voltage relationship but leaves it open for implementation in specific subclasses.

The simplest TwoPinComponent that specifies this relationship is the LipidBilayer:


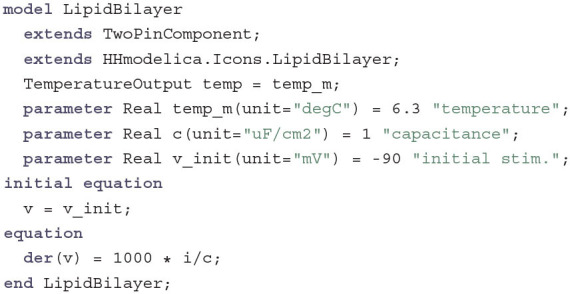


This model inherits the variables and equations of TwoPinComponent and the graphical annotations from the icon LipidBilayer. It therefore only has to introduce one additional equation that describes the component as a capacitor, which separates the inside from the outside and is charged when a current is applied. A factor of 1,000 has to be introduced to measure the derivative der(v) in millivolt instead of volt per second. Since the voltage v only enters the equation as a derivative, the initial value has to be determined. Hodgkin and Huxley used this degree of freedom to simulate a “short initial stimulation” of the Membrane, assuming that it has been kept at a constant *V* = 0 until the time *t* = 0 where the voltage is suddenly changed to *V* = *V*_init_ and is then left to develop under a constant current. In this implementation this behavior is reflected by the parameter v_init. Additionally, the membrane temperature is defined through the parameter temp_m and propagated via the output connector temp.

The temperature is passed on to the Gates, which describe the conformation changes of gating molecules in an ion channel:


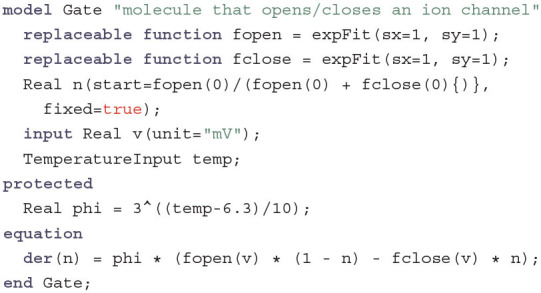


In contrast to the LipidBilayer the Gate does not inherit from TwoPinComponent, since it is not a component in the electric circuit itself but only a part of the IonChannel. Here, n is the gating variable that determines the ratio of gating molecules that are in “open” conformation. The rates with which molecules change formations depend on the current voltage v through the functions fopen, which gives the rate of change from closed to open, and fclose, which gives the rate of change from open to closed. Instead of having variables α and β that change with an equation as in the original formulation by Hodgkin and Huxley, fopen and fclose actually can be seen as variables that store the whole fitting functions. This allows us to keep the code DRY by reusing these functions to determine the starting value for n as the steady state that would be achieved by holding the membrane voltage constant as *V* = 0 mV. In the original model, a change in one of the fitting parameters for the equation for α would also require a change in the stating value for *n* which is not immediately transparent by the description. The functions fopen and fclose are explicitly declared as replaceable so that each ion channel can redefine them as required. For this the three fitting functions expFit, logisticFit and goldmanFit are required in the original HH model. For the sake of simplicity we will only discuss expFit here:


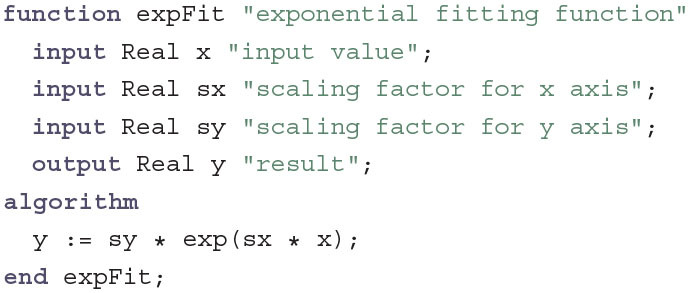


In function definitions, Modelica switches from the usual declarative implementation style to an imperative style as in C or MATLAB. In an algorithm section, equations are variable assignments where an expression on the right-hand side is evaluated and stored in the variable on the left-hand side. This is also indicated by the assignment operator := which has a direction in contrast to the equals sign used for normal equations. During compilation, an algorithm section is transformed to a single equation that depends on all input variables and determines the value of all output variables of the function definition. Here, a simple exponential relationship is defined between the main input x and the output y that can be scaled by the additional fitting parameters sx and sy. These fitting parameters are fixed to a constant value when the function is instantiated as, for example, function fclose = expFit(sx=1, sy=1). The resulting function now has only x left as the single mandatory input and can therefore be called as fclose(x) for any real value x. The functions logisticFit and goldmanFit follow the same general structure, but realize different fitting functions with additional fitting parameters.

As mentioned before, the Gate component is part of an IonChannel component. As there are three different ion channels in the HH model, it again makes sense to introduce a common base class:


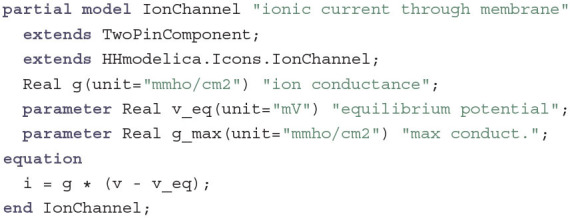


This component is a two-pin component and inherits a graphical annotation from an icon component. It adds the missing relationship between current and voltage by introducing a conductance variable g. If g is constant, the IonChannel behaves as a simple electrical conductor with the only exception that the voltage is relative to the equilibrium potential for the ions transported by this channel. This is true for the LeakChannel which only introduces the additional equation g = g_max. The sodium and potassium channels, however, have voltage- and temperature-dependent gates. Therefore, GatedIonChannel is introduced as another base class that is the same as IonChannel but with an additional TemperatureInput called temp. The delayed-rectifier potassium channel, which lets K^+^ cations pass through the membrane when it is open, then becomes:


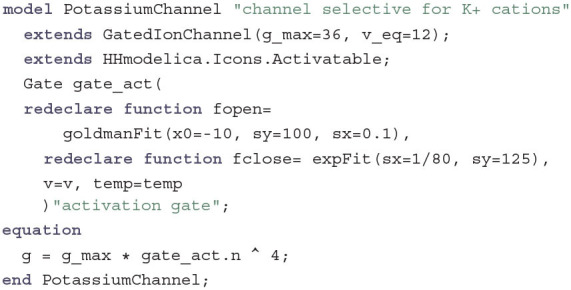


This component inherits variables and equations from GatedIonChannel and at the same time changes the values for the maximum conductance and the equilibrium potential. It uses a Gate component and redeclares the appropriate fitting functions to use for fopen and fclose. The only additional equation introduced is the dependency between the conductance and the gating variable n. As Hodgkin and Huxley determined, four gating molecules have to be in the open conformation simultaneously in order to allow ion transfer through a delayed-rectifier potassium channel. This is realized by taking the fourth power of the gating variable n.

The sodium channel, which lets Na^+^ cations pass through the membrane, looks similar but a little more complex:


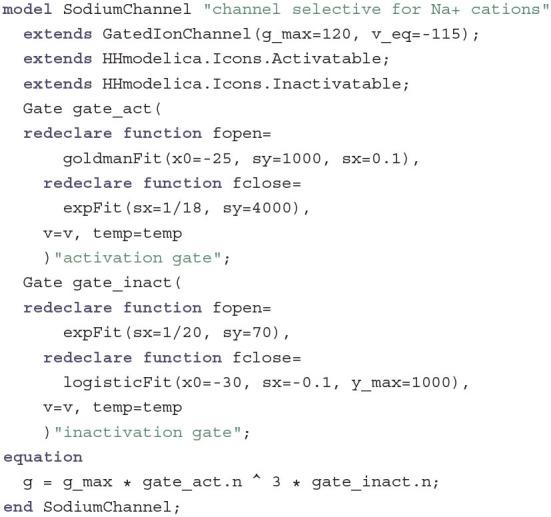


Here we have three molecules that form an activation gate and one molecule that forms an inactivation gate. The gates again only differ in the choice of fitting functions and values for their fitting parameters.

With this we already have all individual components that constitute the cell membrane. To measure and to perform experiments, however, we still need a model of the current clamp which keeps the current through the membrane constant in order to measure the voltage relative to a ground electrode:


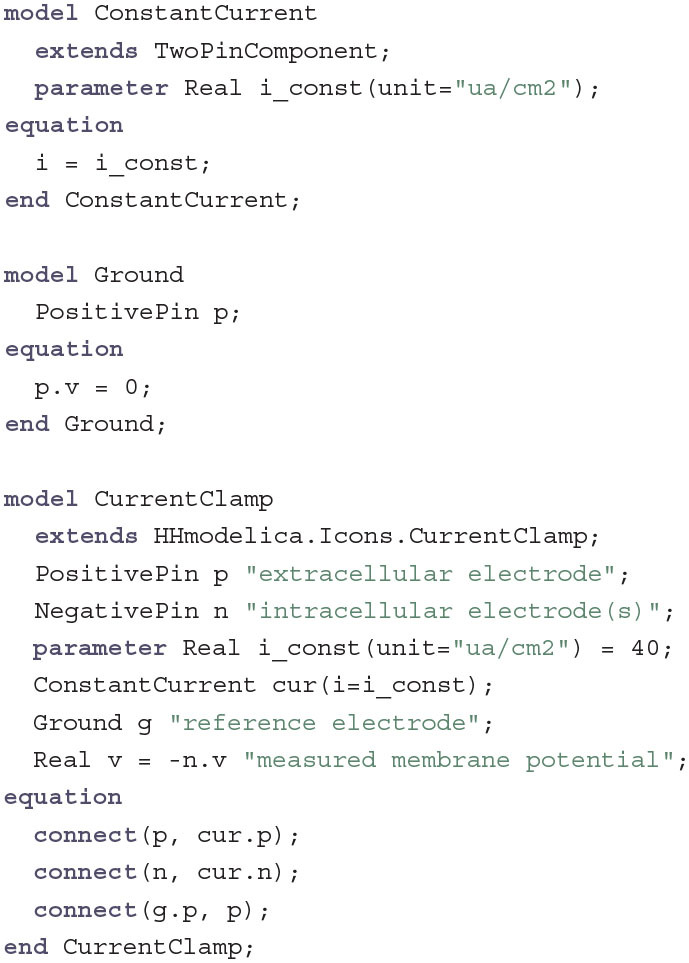


This is the first part in the model where we use Modelica's connect equation to connect smaller components to one large component. The Ground component simply sets the potential of the extracellular compartment to zero while the ConstantCurrent component ensures that the cell has a constant positive outward current. The additional variable v captures the actual membrane potential that would be measured by a real current clamp experiment.

Now that all components are defined, putting together the whole HH model becomes as simple as just placing them side by side, connecting positive with positive and negative with negative pins of neighboring components as well as connecting the TemperatureOutput of the LipidBilayer to all TemperatureInput connectors:


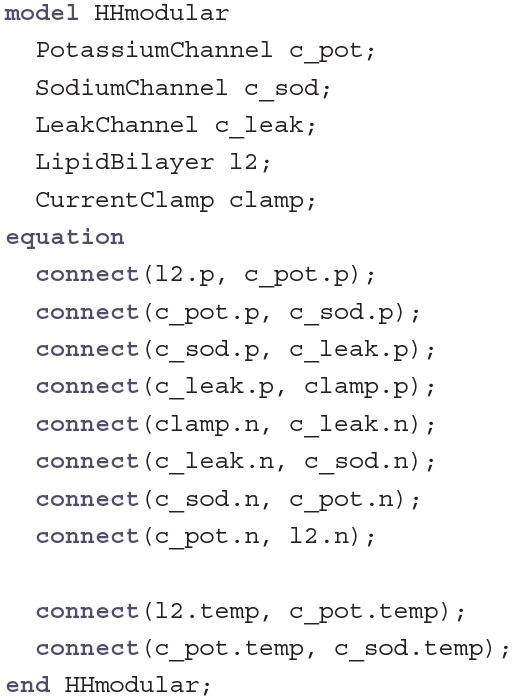


Annotations can be used to place the components on a coordinate system and to give the connections a graphical representation. Usually these annotations are not written manually but generated by an IDE like OpenModelica, where the components can be placed on a diagram view via drag and drop. Due to restrictions in space we do not show all the annotations here but only an example for the placement of the LipidBilayer and the connection between the LipidBilayer and the PotassiumChannel:


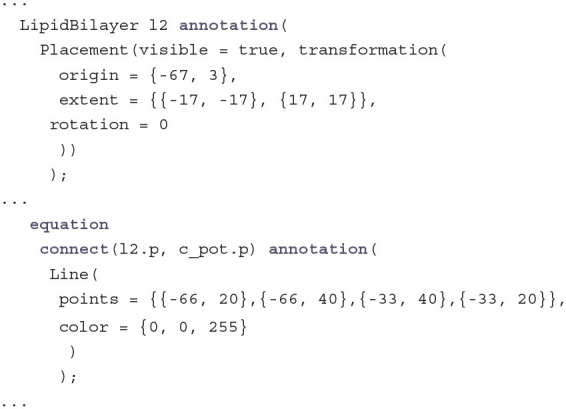


The resulting diagram is shown in Figure 1. The lipid bilayer is represented similar to diagrams in biology textbooks with circles on the outside and curved black lines pointing to the inside of the membrane. The channels are displayed as pores that are either open for the leak channel or closed for channels that have to be activated for ions to pass. The sodium channel additionally has a hinged lid to represent the inactivation gate. Finally, the current clamp is represented by two electrodes piercing the membrane.

### 3.2. Model Validation

**Figure 2:**
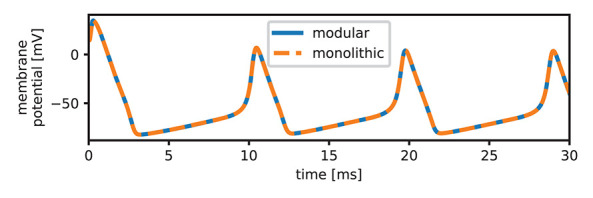
Comparison of the monolithic and modular versions of the HH model. The plot shows perfect alignment of the voltage curves. Note that we plot the membrane potential *V*_*m*_ as difference between the potential on the inside and the potential on the outside of the cell. This conforms with current standards, but is in contrast to the original equations by Hodgkin and Huxley, which define *V* as the displacement from the resting potential with opposite sign. We used the equation *V*_*m*_ = *E*_*r*_ − *V*, assuming a resting potential *E*_*r*_ of −75 mV, which is also used in the HH-implementation in the BioModels database (Le Novère, 2020) and corresponds to the resting potential of the squid giant axon *in vivo* (Moore and Cole, 1960).

When compiled, the modular version of the HH model reduces to the exact same equation system as the original monolithic version. Due to the modularization there are multiple versions of one variable, but this only leads to the addition of a few trivial equations of the form *x* = *y* or the form *x* = −*y*. Figure 2 shows a plot of the monolithic and modular version to ensure that both are functionally equivalent. This means that there are now two very different implementations with the same functionality which can and have to be analyzed for their suitability according to the research questions we established in our introduction.

### 3.3. Assessment of Understandability (RQ1)

With RQ1, we asked if the understandability of the HH model can be improved by a modular implementation that bridges the gap between biological meaning and electrical analogy. Using cognitive load theory as a framework, we will assume that a model is more understandable if it requires fewer items to be kept in working memory simultaneously. Although the modular implementation has more lines of code in total, it separates the model into small digestible parts. When a novice, for example, wants to know what a Gate is, they only have to process five variables and one equation, each of which are documented with their physiological meaning. When they understand this component, they know that its purpose is to produce a value between zero and one which is based on voltage and temperature and represents the ratio of gating molecules in open conformation. Once this concept is stored in long-term memory it can be recalled as a single item into working memory. This means that, when the learner moves on, the two gates in SodiumChannel can be processed as two items instead of twelve^2^. Moving from component to component, the modular version presents the reader with at most two equations and five variables or parameters at the same time. This constitutes a very low cognitive load compared to the 15 equations and 33 variables and parameters of the monolithic version that are presented all at once or in loose groups without clearly defined interfaces.

One way to facilitate the transfer of new concepts in long-term memory is to anchor them to existing knowledge. Our implementation does this by annotating each component and each variable or parameter in that component with biological terms^3^. The implementation also uses speaking variable names wherever possible to keep a close link between the biological and the mathematical representation—a common best practice in software engineering (that could also be applied to a monolithic version). Some parts of the model, such as the seemingly arbitrary fitting function goldmanFit, require more explanation which can be given in Modelica by adding an HTML string to the component for a detailed documentation.

Finally, on the highest hierarchical level, the component HHmodular has still only five variables but ten connect equations. For this model, however, a novice does not need to read any code at all to understand it, because they can use the diagram in Figure 1 instead. Since it is defined directly in the code and tied to the individual components it is not only a simplified documentation but an accurate graphical reflection of the underlying implementation. This means that understanding the model on this level of abstraction is not any harder than understanding a corresponding biological drawing in a textbook. Assuming that the learner is already familiar with *what* is modeled, the implementation can again be anchored easily to that existing knowledge.

### 3.4. Assessment of Extensibility and Reusability (RQ2)

With RQ2, we asked if a modular implementation of the HH model can serve as a unifying basis for extensions and therefore facilitate the creation of more complex HH-type modules. The current implementation already could reduce some duplicate equations in the original model by reusing already existing code. The current-voltage relationship of the three ion channels, the rate of conformation change in the three different gates, and the fitting functions expFit and goldmanFit each had to be defined only once. Furthermore, the introduction of the replaceable functions fopen and fclose eliminated the need to define starting values for the gating variables. When new components are added to the model, it is highly probable that some of these existing components can be used to reduce the implementation effort. Even more importantly, the argument for the reduction of cognitive load by modularization gets more weight as the model grows in size. In the modular version, the only point where cognitive load may increase due to extensions and therefore make the model less understandable is when there are too many individual models at the highest hierarchical layer. However, even then it is possible to form groups of components (e.g., a group for all potassium-sensitive channels or for all ion pumps) that are then connected on a new even higher hierarchical level. Conversely, the cognitive load associated with a monolithic model will grow with each variable and each equation that is added to the model.

To give one specific example, a reasonable extension could be the inclusion of slow inactivation of sodium channels. In contrast to fast inactivation, that stops the influx of Na^+^ cations after a few milliseconds, slow inactivation takes place over seconds or even minutes of prolonged or high frequency depolarizations, reducing the number of sodium channels available for activation (Payandeh, 2018). This could be realized by simply adding another Gate component to the SodiumChannel and introducing a ratio p_slow that determines how much of the total current is attributed to slow as opposed to fast inactivation. The conductance equation would then change from


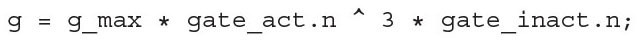


to:


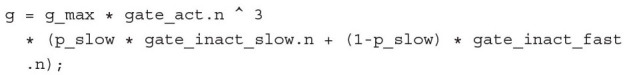


Apart from choosing appropriate fitting functions for the new gate, this would be the only change required. Arguably a monolithic model would not require more changes, but it would be more difficult to first identify which equations have to change and thus it would be easier to make a mistake by missing or interchanging an equation or variable. We encountered this problem in a previous work with a model of the human cardiac conduction system (Schölzel et al., 2020).

Other extensions might involve defining an alternative Gate that uses fitting functions to determine the steady state *n*_∞_ and time constant τ instead of α (fopen) and β (fclose) (Destexhe and Huguenard, 2000; Goldman et al., 2001) or new components based on TwoPinComponent, such as channel formulations based on the Goldman-Hodgkin-Katz flux equation (Huguenard and McCormick, 1992; Destexhe and Huguenard, 2000) or models of ionic pumps (Di Francesco and Noble, 1985; Matsuoka and Hilgemann, 1992). Even in these cases the underlying interfaces can stay the same and parts like the current clamp formulation, common base classes, and fitting functions can be reused.

## 4. Discussion

We showed that a modular version of the HH model that uses software-engineering techniques to manage complexity is beneficial both for novices and for experts, answering both of our research questions in the affirmative.

RQ1 asked whether the understandability of the HH model can be improved by a modular implementation. We showed that this is the case using CLT as framework and demonstrating a drop of the cognitive load by a factor of 6. The biological concepts can be explained and understood one at a time with an accurate graphical representation at the highest level of abstraction instead of having to navigate through a multitude of equations and variables with high element interactivity. In summary this means that with our implementation a deeper understanding of the HH model can be achieved in less time and it is likely that novices learning the model in this way will make fewer errors when recalling the learned concepts at a later time.

RQ2 asked whether the modular implementation can also serve as a unifying basis for extensions and facilitate the creation of more complex HH-type models. We showed that, in contrast to the monolithic version, adding new components does not significantly increase the cognitive load associated with the model. We also demonstrated that many components of our model are easily reusable which reduces development time and increases interoperability of solutions.

Similar results like ours would have been possible, for example, using CellML, or SBML with the SBML-comp package. In fact, Wimalaratne et al. (2009) also used the Hodgkin-Huxley model as an example to promote the support for hierarchical composition of CellML models. However, we used some Modelica features for our design that do not exist in these other languages. This includes the graphical composition of models, object-oriented programming with multiple inheritance, acausal connections between electrical and chemical components, the grouping of interface variables to connectors, and the annotation of the experiment setup within the model file itself.

One limitation of this approach is that some experts might be much more familiar with the formalism of differential equations than with object-oriented software design. It might be easier for them to reduce a group of equations to a single item in their working memory than it is to do the same with a piece of code that represents a class. This means that, for models of small to moderate size, the navigation through different classes according to a modular design structure might actually be detrimental to their understanding of the model and to their productivity when working with it. A solution to this problem could be to provide a third, equation-based representation of the respective model in parallel to the existing graphical view and the raw code. Authoring tools like OpenCOR for CellML (Garny and Hunter, 2015) or COPASI for SBML (Hoops et al., 2006) already provide these equation-based views. However, they do not provide an overview of all equations in the whole model, but only of the parts that are currently selected. For Modelica we are only aware of a similar approach to OpenCOR and COPASI in the proprietary IDE MapleSim (Maplesoft, 2020). Implementing such a representation in open-source tools, such as OpenModelica would be possible due to the fact that, like CellML and SBML, Modelica is declarative. OpenModelica already has a feature to “instantiate” a model, which reduces its structure to a “flat” format consisting of a single class with a list of parameters, variables, and equations. Additionally, OpenModelica models can be exported in an XML format that contains all parameters, variables, and equations in a machine-readable form. Based on these existing features, an “equation view” could be implemented in the OpenModelica IDE OMEdit that would allow experts to understand a model at first glance based on the differential equations and without having to traverse its hierarchical structure. Alternatively, such a representation could be part of a documentation website associated with a model or model library. As an added benefit a tool that provides such an automated representation as typeset equations could also provide an export as LaTeX or Word documents, which can then be inserted in articles to guarantee that the published version of the equations is exactly the same as the equations used to simulate the model. We have implemented a first prototype of such an equation-based web documentation using the Julia package Documenter.jl (Piibeleht et al., 2020) and our own package ModelicaScriptingTools.jl (Schölzel, 2020). The resulting experimental documentation for the Hodgkin-Huxley model presented in this article can be found at https://cschoel.github.io/hh-modelica/dev/.

Another direction for future research is the application of our techniques to larger and more complex models. We are already using the model developed in this paper as a basis to reproduce a large 116 equation model of the atrioventricular node (Inada et al., 2009). We hope that this and other projects based on the same methodology, be it with Modelica or another MoDROGH language like CellML, may help to increase the quality and speed of scientific progress in systems biology.

## Data Availability Statement

The original contributions presented in the study are publicly available. This data can be found here: https://github.com/CSchoel/hh-modelica (archived as https://doi.org/10.5281/zenodo.3947848).

## Author Contributions

CS and AD conceived the project. CS implemented the models and performed the experiments. VB and GE provided the physiological consultation and critique. CS drafted the manuscript. All authors contributed to manuscript revision, read, and approved the submitted version.

## Conflict of Interest

The authors declare that the research was conducted in the absence of any commercial or financial relationships that could be construed as a potential conflict of interest.

## Footnotes

^1^For a comparison of different language candidates see the supplementary note in Schölzel et al. (2020).

^2^This benefit is even more pronounced considering that the formulas for the α and β variables in the original model each consist up to seven seemingly arbitrary mathematical operations that are now given a meaning by introducing the named and documented fitting functions expFit, logisticFit, and goldmanFit.

^3^Due to spatial limitations we do not show all these annotations in this paper, but they can be found at https://github.com/CSchoel/hh-modelica/.
