# Supplementary material for: An Understandable, Extensible, and Reusable Implementation of the Hodgkin-Huxley Equations Using Modelica
Source: Front Physiol. 2020 Oct 2;11:583203. doi: 10.3389/fphys.2020.583203 (PMC7566415; doi:10.3389/fphys.2020.583203)
Supplement: Supplementary file 1 [file Data_Sheet_1.pdf]

frontiers

Data Supplement

An understandable, extensible and reusable implementation of the Hodgkin-Huxley equations using Modelica

Christopher Schölzel, Valeria Blesius, Gernot Ernst, and Andreas Dominik
Thursday 17th September, 2020

RATIONALE FOR PUBLISHING CODE AS PDF

This data supplement contains the full model code. This code is also available on GitHub[1], which is the preferred source since it may contain fixes and updates applied after the publication of this paper. However, GitHub may not always be there in the future and therefore we provide this version to be archived along with the paper.

PACKAGE STRUCTURE

Modelica organizes code in packages. These packages can be defined within a single file or, in a folder structure where each folder contains a file called `package.mo` that contains the package metadata. This project uses a mix of both styles: Since the components themselves are very small, they are grouped in the single file `HHModelica/Components/package.mo`. Full models and Icons, however, are created in their own separate files.

PACKAGE METADATA

Listing 1: `HHmodelica/package.mo`

```
package HHmodelica "Modelica implementations of the Hogdkin-Huxley model"
end HHmodelica;
```

Listing 2: `HHmodelica/CompleteModels/package.mo`

```
within HHmodelica;
package CompleteModels
end CompleteModels;
```

Listing 3: `HHmodelica/Icons/package.mo`

```
within HHmodelica;
package Icons "contains icon classes that are used to inherit annotations"
end Icons;
```

COMPONENTS

Listing 4: `HHmodelica/Components/package.mo`

```
within HHmodelica;
package Components "components for the two-pin modular version of the
   Hodgkin-Huxley model"
  connector TemperatureInput = input Real(unit="degC") "membrane
     temperature"
    annotation(
      Icon(
        coordinateSystem(
          preserveAspectRatio=true,
          extent={{-100,-100},{100,100}}
        ),
        graphics={
          Ellipse(
            extent={{-100,100},{100,-100}},
```

[1] https://github.com/CSchoel/hh-modelica

```
          lineColor={255,0,0},
          fillColor={255,255,255},
          fillPattern=FillPattern.Solid
        )
      }
    )
  );
  connector TemperatureOutput = output Real(unit="degC") "membrane
     temperature"
    annotation(
      Icon(
        coordinateSystem(
          preserveAspectRatio=true,
          extent={{-100,-100},{100,100}}
        ),
        graphics={
          Ellipse(
            extent={{-100,100},{100,-100}},
            lineColor={255,0,0},
            fillColor={255,0,0},
            fillPattern=FillPattern.Solid
          )
        }
      )
    );

  connector ElectricalPin "electrical connector for membrane currents"
    flow Real i(unit="uA/cm2") "ionic current through membrane";
    Real v(unit="mV") "membrane potential (as displacement from resting
       potential)";
  end ElectricalPin;

  connector PositivePin "electrical pin with filled square icon for visual
      distinction"
    extends ElectricalPin;
    annotation(
      Icon(
        coordinateSystem(
          preserveAspectRatio=true,
          extent={{-100,-100},{100,100}}
        ),
        graphics={
          Rectangle(
            extent={{-100,100},{100,-100}},
            lineColor={0,0,255},
            fillColor={0,0,255},
            fillPattern=FillPattern.Solid
          )
        }
      )
    );
  end PositivePin;

  connector NegativePin "electrical pin with open square icon for visual
     distinction"
    extends ElectricalPin;
    annotation(
      Icon(
        coordinateSystem(
          preserveAspectRatio=true,
          extent={{-100,-100},{100,100}}
        ),
```

```
        graphics={
          Rectangle(
            extent={{-100,100},{100,-100}},
            lineColor={0,0,255},
            fillColor={255,255,255},
            fillPattern=FillPattern.Solid
          )
        }
      )
    );
  end NegativePin;

  partial model TwoPinComponent "component with two directly connected
     electrical pins"
    PositivePin p "positive extracellular pin" annotation (Placement(
       transformation(extent={{-10, 90},{10, 110}})));
    NegativePin n "negative intracellular pin" annotation (Placement(
       transformation(extent={{-10, -90},{10, -110}})));
    Real v(unit="mV") "voltage as potential difference between positive
       and negative pin";
    Real i(unit="uA/cm2") "outward current flowing through component from
       negative to positive pin";
  equation
    i = p.i;
    0 = p.i + n.i;
    v = p.v - n.v;
    annotation(
      Documentation(info="
        <html>
          <p>This is a base model for components with two electrical pins
             which
          are directly connected. It establishes the connection and
             defines a
          voltage between the positive and negative pin, but does not
             specify
          the current-voltage relationship.</p>
          <p>This base component establishes the convention that positive
          currents are outward currents and negative currents are inward
          currents.</p>
        </html>
      ")
    );
  end TwoPinComponent;

  function expFit "exponential function with scaling parameters for x and
     y axis"
    input Real x "input value";
    input Real sx "scaling factor for x axis (fitting parameter)";
    input Real sy "scaling factor for y axis (fitting parameter)";
    output Real y "result";
  algorithm
    y := sy * exp(sx * x);
  end expFit;

  function goldmanFit "fitting function related to Goldmans formula for
     the movement of a charged particle in a constant electrical field"
    input Real x "membrane potential (as displacement from resting
       potential)";
    input Real x0 "offset for x (fitting parameter)";
    input Real sx "scaling factor for x (fitting parameter)";
    input Real sy "scaling factor for y (fitting parameter)";
    output Real y "rate of change of the gating variable at given V=x";
```

```
protected
  Real x_adj "adjusted x with offset and scaling factor";
algorithm
  x_adj := sx * (x - x0);
  if abs(x - x0) < 1e-6 then
    y := sy; // using L'Hôpital to find limit for x_adj->0
  else
    y := sy * x_adj / (exp(x_adj) - 1);
  end if;
  annotation(
    Documentation(info="
      <html>
        <p>Hodgkin and Huxley state that this formula was (in part) used
        because it &quot;bears a close resemblance to the equation
           derived
        by Goldman (1943) for the movements of a charged particle in a
           constant
        field&quot;.</p>
        <p>We suppose that this statement refers to equation 11 of
           Goldman
        (1943), which is also called the Goldman-Hodgkin-Katz flux
           equation:</p>
        <blockquote>
          j_i = u_i * F / a * dV * (n'_i * exp(-z_i * beta * dV - n0_i))
              / exp(-z_i * beta * dV)
        </blockquote>
        <p>Factoring out n0_i from the denominator, substituting
        n'_i/n0_i = exp(V_0 * beta * z_i) and grouping and renaming
        variables, the GHK flux equation can be written as</p>
        <blockquote>
          y := sy * sx * x * (exp((x - x0) * sx) - 1) / (exp(x * sx) -
             1)
        </blockquote>
        <p>with sx = -z_i * beta, x = dV, x0 = V_0, and
        sy = n0_i * u_i * F / a * 1 / sx.</p>
        <p>With this notation, the similarity becomes apparent, as
           omitting
        the denominator (exp((x - x0) * sx) - 1) and using x-x0 instead
           of
        x in the rest of the formula gives exactly the goldmanFit used
           by
        Hodgkin and Huxley.</p>
      </html>
    ")
  );
end goldmanFit;

function logisticFit "logistic function with sigmoidal shape"
  input Real x "input value";
  input Real x0 "x-value of sigmoid midpoint (fitting parameter)";
  input Real sx "growth rate/steepness (fitting parameter)";
  input Real y_max "maximum value";
  output Real y "result";
protected
  Real x_adj "adjusted x with offset and scaling factor";
algorithm
  x_adj := sx * (x - x0);
  y := y_max / (exp(-x_adj) + 1);
end logisticFit;

model Gate "gating molecule with an open conformation and a closed
   conformation"
```

```
    replaceable function fopen = expFit(sx=1, sy=1) "rate of transfer from
        closed to open conformation";
    replaceable function fclose = expFit(sx=1, sy=1) "rate of transfer
       from open to closed conformation";
    Real n(start=fopen(0)/(fopen(0) + fclose(0)), fixed=true) "ratio of
       molecules in open conformation";
    input Real v(unit="mV") "membrane potential (as displacement from
       resting potential)";
    TemperatureInput temp "membrane temperature";
  protected
    Real phi = 3^((temp-6.3)/10) "temperature-dependent factor for rate of
        transfer calculated with Q10 = 3";
  equation
    der(n) = phi * (fopen(v) * (1 - n) - fclose(v) * n);
  end Gate;

  partial model IonChannel "ionic current through the membrane"
    extends TwoPinComponent;
    extends HHmodelica.Icons.IonChannel;
    Real g(unit="mmho/cm2") "ion conductance, needs to be defined in
       subclasses";
    parameter Real v_eq(unit="mV") "equilibrium potential (as displacement
        from resting potential)";
    parameter Real g_max(unit="mmho/cm2") "maximum conductance";
  equation
    i = g * (v - v_eq);
  end IonChannel;

  partial model GatedIonChannel "ion channel that has voltage-dependent
     gates"
    extends IonChannel;
    TemperatureInput temp "membrane temperature to determine reaction
       coefficient"
      annotation (Placement(transformation(extent={{-40, 48},{-60, 68}})))
        ;
  end GatedIonChannel;

  model PotassiumChannel "channel selective for K+ cations"
    extends GatedIonChannel(g_max=36, v_eq=12);
    extends HHmodelica.Icons.Activatable;
    Gate gate_act(
      redeclare function fopen= goldmanFit(x0=-10, sy=100, sx=0.1),
      redeclare function fclose= expFit(sx=1/80, sy=125),
      v=v, temp=temp
    ) "activation gate";
  equation
    g = g_max * gate_act.n ^ 4;
  end PotassiumChannel;

  model SodiumChannel "channel selective for Na+ cations"
    extends GatedIonChannel(g_max=120, v_eq=-115);
    extends HHmodelica.Icons.Activatable;
    extends HHmodelica.Icons.Inactivatable;
    Gate gate_act(
      redeclare function fopen= goldmanFit(x0=-25, sy=1000, sx=0.1),
      redeclare function fclose= expFit(sx=1/18, sy=4000),
      v=v, temp=temp
    ) "activation gate";
    Gate gate_inact(
      redeclare function fopen= expFit(sx=1/20, sy=70),
      redeclare function fclose= logisticFit(x0=-30, sx=-0.1, y_max=1000),
      v=v, temp=temp
```

```
    ) "inactivation gate";
  equation
    g = g_max * gate_act.n ^ 3 * gate_inact.n;
  end SodiumChannel;

  model LeakChannel "constant leakage current of ions through membrane"
    extends IonChannel(g_max=0.3, v_eq=-10.613);
    extends HHmodelica.Icons.OpenChannel;
  equation
    g = g_max;
  end LeakChannel;

  model LipidBilayer "lipid bilayer separating external and internal
     potential (i.e. acting as a capacitor)"
    extends TwoPinComponent;
    extends HHmodelica.Icons.LipidBilayer;
    TemperatureOutput temp = temp_m annotation (Placement(transformation(
       extent={{40, 48},{60, 68}})));
    parameter Real temp_m(unit="degC") = 6.3 "constant membrane
       temperature";
    parameter Real c(unit="uF/cm2") = 1 "membrane capacitance";
    parameter Real v_init(unit="mV") = -90 "short initial stimulation";
  initial equation
    v = v_init;
  equation
    der(v) = 1000 * i / c "multiply with 1000 to get mV/s instead of v/s";
  end LipidBilayer;

  model ConstantCurrent "applies current to positive pin regardless of
     voltage"
    extends TwoPinComponent;
    parameter Real i_const(unit="uA/cm2") "current applied to positive pin
       ";
  equation
    i = i_const;
  end ConstantCurrent;

  model Ground "sets voltage to zero, acting as a reference for measuring
     potential"
    PositivePin p;
  equation
    p.v = 0;
  end Ground;

  model Membrane "full membrane model that can be used in current clamp
     experiments"
    extends HHmodelica.Icons.LipidBilayer;
    PositivePin p "positive extracellular pin" annotation (Placement(
       transformation(extent={{-10, 90},{10, 110}})));
    NegativePin n "negative intracellular pin" annotation (Placement(
       transformation(extent={{-10, -90},{10, -110}})));
    PotassiumChannel c_pot;
    SodiumChannel c_sod;
    LeakChannel c_leak;
    LipidBilayer l2 "lipid bilayer as capacitor";
  equation
    connect(c_pot.p, l2.p);
    connect(c_pot.n, l2.n);
    connect(c_sod.p, l2.p);
    connect(c_sod.n, l2.n);
    connect(c_leak.p, l2.p);
    connect(c_leak.n, l2.n);
```

```
    connect(p, l2.p);
    connect(n, l2.n);
    connect(c_pot.temp, l2.temp);
    connect(c_sod.temp, l2.temp);
  end Membrane;

  model CurrentClamp "current clamp that applies constant current to the
     membrane"
    extends HHmodelica.Icons.CurrentClamp;
    PositivePin p "extracellular electrode" annotation (Placement(
       transformation(extent={{-10, 90},{10, 110}})));
    NegativePin n "intracellular electrode(s)" annotation (Placement(
       transformation(extent={{-10, -90},{10, -110}})));
    parameter Real i_const(unit="uA/cm2") = 40 "current applied to
       membrane";
    ConstantCurrent cur(i_const=i_const) "external current applied to
       membrane";
    Ground g "reference electrode";
    Real v(unit="mV") = -n.v "measured membrane potential";
  equation
    connect(p, cur.p);
    connect(n, cur.n);
    connect(g.p, p);
  end CurrentClamp;

end Components;
```

COMPLETE MODELS

Listing 5: HHmodelica/CompleteModels/HHmono.mo

```
within HHmodelica.CompleteModels;
partial model PotentialAdapter "base class that converts membrane
   potential to current standards"
  parameter Real e_r(unit="mV") = -75 "resting potential";
  Real v_m(unit="mV") = e_r - v "absolute membrane potential (v_in - v_out
     )";
  Real v(unit="mV") "membrane potential as displacement from resting
     potential (out - in)";
  annotation(
    experiment(StartTime = 0, StopTime = 30, Tolerance = 1e-6, Interval =
       0.01),
    __OpenModelica_simulationFlags(s = "dassl"),
    __MoST_experiment(variableFilter="v_m|v|gK|gNa|n|m|h"),
    Documentation(info="
      <html>
        <p>The variable e_r in this adapter can be used to plot the
           absolute
        membrane potential as difference between the potential on the
           inside
        and the potential on the outside of the cell.
        This conforms with current standards, but not to the original
           equations
        by Hodgkin and Huxley, which define V as the displacement from the
        resting potential with opposite sign.</p>
        <p>For this conversion, a value for the resting potential e_r must
            be
        assumed, which is not given in the original article. We use e_r =
           -75 mV,
        because this is the value that is used by the BioModels
           implementation of
        the Hodgkin-Huxley model and corresponds to the resting potential
```

```
        measured for the squid giant axon <i>in vivo</i>
        (cf. Moore and Cole, 1960, https://doi.org/10.1085/jgp.43.5.961)
          .</p>
      </html>
    ")
  );
end PotentialAdapter;
```

Listing 6: `HHmodelica/CompleteModels/HHmono.mo`

```
within HHmodelica.CompleteModels;
model HHmono "monolithic version of the Hodgkin-Huxley model"
  extends PotentialAdapter;
  parameter Real e_r(unit="mV") = -75 "resting potential";
  Real v_m(unit="mV") = e_r - v "absolute membrane potential (v_in - v_out
    )";
  parameter Real Cm(unit = "uF/cm2")        = 1;
  parameter Real gbarNa(unit = "mmho/cm2")    = 120 "max sodium conductance
    ";
  parameter Real gbarK(unit = "mmho/cm2")     = 36 "max potassium
    conductance";
  parameter Real gbar0(unit = "mmho/cm2")     = 0.3;
  parameter Real VNa(unit = "mV")       = -115;
  parameter Real VK(unit = "mV")        = 12;
  parameter Real Vl(unit = "mV")        = -10.613;
  parameter Real Temp     = 6.3 ;
  parameter Real phi      = 3^((Temp-6.3)/10);
  parameter Real Vdepolar(unit = "mV") = -90;
  parameter Real Vnorm(unit = "mV")     = 1 "for non-dimensionalizing v in
    function expressions, i.e. exp(v/Vnorm) replaces exp(v).";
  parameter Real msecm1(unit = "1/msec") = 1 "for adding units to alpha
    and beta variables";
  parameter Real alphan0 (unit="1/msec") = 0.1/(exp(1)-1) "always use v=0
    to calculate i.c.";
  parameter Real betan0  (unit="1/msec") = 0.125;
  parameter Real alpham0 (unit="1/msec") = 2.5/(exp(2.5)-1);
  parameter Real betam0  (unit="1/msec") = 4;
  parameter Real alphah0 (unit="1/msec") = 0.07;
  parameter Real betah0  (unit="1/msec") = 1/(exp(3)+1);

  parameter Real minusI(unit = "nA/cm2") = 40;
  input Real Vclamp(unit = "mV");

  parameter Real clamp_0no_1yes = 0;

  //Variables for all the algebraic equations
  Real INa(unit = "nA/cm2") "Ionic currents";
  Real IK(unit = "nA/cm2") "Ionic currents";
  Real Il(unit = "nA/cm2") "Ionic currents";
  Real alphan(unit = "1/msec")  "rate constant of particles from out to in
    ";
  Real betan(unit = "1/msec")   "rate constant from in to out";
  Real alpham(unit = "1/msec")  "rate constant of activating molecules
    from out to in";
  Real betam(unit = "1/msec")   "rate constant of activating molecules
    from in to out";
  Real alphah(unit = "1/msec")  "rate constant of inactivating molecules
    from out to in";
  Real betah(unit = "1/msec")   "rate constant of inactivating molecules
    from in to out";
  Real gNa(unit = "mmho/cm2")   "Sodium conductance";
  Real gK(unit = "mmho/cm2")    "potassium conductance";
```

```
  Real Iion(unit = "nA/cm2");

  //State variables for all the ODEs
  Real VV(unit="mV");
  Real v(unit="mV") "displacement of the membrane potential from its
     resting value (depolarization negative)";
  Real n "proportion of the particles in a certain position";
  Real m "proportion of activating molecules on the inside";
  Real h "proportion of inactivating molecules on the outside";

protected
  Real ninf;
  Real minf;
  Real hinf;
  Real taun(unit="msec");
  Real taum(unit="msec");
  Real tauh(unit="msec");

initial equation
  VV = if clamp_0no_1yes == 0 then Vdepolar else Vclamp;

  n = alphan0/(alphan0+betan0);
  m = alpham0/(alpham0+betam0);
  h = alphah0/(alphah0+betah0);

equation
  //if v/Vnorm == -10 then
  //  alphan = 0.1;
  //else
  alphan = 0.01 * (v / Vnorm + 10) / (exp((v / Vnorm + 10) / 10) - 1);
  //end if;
  betan = msecm1 * (0.125 * exp(v / Vnorm / 80));
  //if v/Vnorm == -25 then
  //  alpham = 1;
  //else
  alpham = 0.1 * (v / Vnorm + 25) / (exp((v / Vnorm + 25) / 10) - 1);
  //end if;
  betam = msecm1 * (4 * exp(v / Vnorm / 18));
  alphah  = msecm1*(0.07*exp((v/Vnorm)/20));
  betah   = msecm1*(1/(exp(( v/Vnorm+30)/10)+1));
  minf    = alpham/(alpham+betam);
  ninf    = alphan/(alphan+betan);
  hinf    = alphah/(alphah+betah);
  taun    =1/(alphan+betan);
  tauh    =1/(alphah+betah);
  taum    =1/(alpham+betam);
  gNa     = gbarNa * m^3 * h;
  gK      = gbarK  * n^4;
  INa     = gNa * (v-VNa);
  IK      = gK  * (v-VK);
  Il      = gbar0 * (v-Vl);
  Iion    = INa + IK + Il;
  if (clamp_0no_1yes == 0) then
    der(VV) = (-minusI-INa-IK-Il)/Cm;
    v = VV;
  else
    der(VV) = 0;
    v = Vclamp;
  end if;
  der(n) = phi*(alphan*(1-n)-betan*n);
  der(m) = phi*(alpham*(1-m)-betam*m);
  der(h) = phi*(alphah*(1-h)-betah*h);
```

```
annotation(
  experiment(StartTime = 0, StopTime = 30, Tolerance = 1e-6, Interval =
     0.01),
  __OpenModelica_simulationFlags(s = "dassl"),
  __MoST_experiment(variableFilter="v_m|v|gK|gNa|n|m|h")
);
end HHmono;
```

Listing 7: `HHmodelica/CompleteModels/HHmodular.mo`

```
within HHmodelica.CompleteModels;
model HHmodular "'flat' version of the modular model (no membrane
    container)"
  extends PotentialAdapter(v = l2.v);
  HHmodelica.Components.PotassiumChannel c_pot annotation(
    Placement(visible = true, transformation(origin = {-33, 3}, extent =
       {{-17, -17}, {17, 17}}, rotation = 0)));
  HHmodelica.Components.SodiumChannel c_sod annotation(
    Placement(visible = true, transformation(origin = {1,3}, extent =
       {{-17, -17}, {17, 17}}, rotation = 0)));
  HHmodelica.Components.LeakChannel c_leak annotation(
    Placement(visible = true, transformation(origin = {35, 3}, extent =
       {{-17, -17}, {17, 17}}, rotation = 0)));
  HHmodelica.Components.LipidBilayer l2 annotation(
    Placement(visible = true, transformation(origin = {-67, 3}, extent =
       {{-17, -17}, {17, 17}}, rotation = 0)));
  HHmodelica.Components.CurrentClamp clamp annotation(
    Placement(visible = true, transformation(origin = {69, 3}, extent =
       {{-17, -17}, {17, 17}}, rotation = 0)));
equation
  connect(l2.p, c_pot.p) annotation(
    Line(points = {{-66, 20}, {-66, 40}, {-33, 40}, {-33, 20}}, color =
       {0, 0, 255}));
  connect(c_pot.p, c_sod.p) annotation(
    Line(points = {{-33, 20}, {-32, 20}, {-32, 40}, {0, 40}, {0, 20}, {2,
       20}}, color = {0, 0, 255}));
  connect(c_sod.p, c_leak.p) annotation(
    Line(points = {{2, 20}, {2, 20}, {2, 40}, {34, 40}, {34, 20}, {36,
       20}}, color = {0, 0, 255}));
  connect(c_leak.p, clamp.p) annotation(
    Line(points = {{36, 20}, {36, 20}, {36, 40}, {68, 40}, {68, 20}, {70,
       20}}, color = {0, 0, 255}));
  connect(clamp.n, c_leak.n) annotation(
    Line(points = {{70, -14}, {68, -14}, {68, -40}, {36, -40}, {36, -14},
       {36, -14}}, color = {0, 0, 255}));
  connect(c_leak.n, c_sod.n) annotation(
    Line(points = {{36, -14}, {34, -14}, {34, -40}, {2, -40}, {2, -14},
       {2, -14}}, color = {0, 0, 255}));
  connect(c_sod.n, c_pot.n) annotation(
    Line(points = {{2, -14}, {0, -14}, {0, -40}, {-32, -40}, {-32, -14},
       {-33, -14}}, color = {0, 0, 255}));
  connect(c_pot.n, l2.n) annotation(
    Line(points = {{-33, -14}, {-33, -40}, {-66, -40}, {-66, -14}}, color
       = {0, 0, 255}));
  connect(l2.temp, c_pot.temp) annotation(
    Line(points = {{-58, 12}, {-58, 16}, {-42, 16}, {-42, 13}}, color =
       {255, 0, 0}));
  connect(c_pot.temp, c_sod.temp) annotation(
    Line(points = {{-42, 13}, {-40, 13}, {-40, 16}, {-8, 16}, {-8, 12}},
       color = {255, 0, 0}));
annotation(
```

```
  experiment(StartTime = 0, StopTime = 0.03, Tolerance = 1e-6, Interval =
     1e-05),
  __OpenModelica_simulationFlags(s = "dassl"),
  __MoST_experiment(variableFilter="v_m|clamp\\.(v|i)|c_pot\\.(g|gate_act
     \\.n)|c_sod\\.(g|gate_act\\.n|gate_inact\\.n)")
);
end HHmodular;
```

Listing 8: `HHmodelica/CompleteModels/HHmodHier.mo`

```
within HHmodelica.CompleteModels;
model HHmodHier
  extends PotentialAdapter(v = m.l2.v);
  import HHmodelica.Components.Membrane;
  import HHmodelica.Components.CurrentClamp;
  Membrane m;
  // i = 40 => recurring depolarizations
  // i = 0 => v returns to 0
  CurrentClamp c(i_const=40);
equation
  connect(m.p, c.p);
  connect(m.n, c.n);
annotation(
  experiment(StartTime = 0, StopTime = 0.03, Tolerance = 1e-6, Interval =
     1e-05),
  __OpenModelica_simulationFlags(s = "dassl"),
  __MoST_experiment(variableFilter="c\\.(v|i)|m\\.c_pot\\.(g|gate_act\\.n)
     |m\\.c_sod\\.(g|gate_act\\.n|gate_inact\\.n)")
);
end HHmodHier;
```

ICONS

Listing 9: `HHmodelica/Icons/LipidBilayer.mo`

```
within HHmodelica.Icons;
model LipidBilayer "lipid bilayer with red circles on outside and black
    lines on inside"
annotation(
  Icon(
    coordinateSystem(
      preserveAspectRatio= false,
      extent= {{-100,-100},{100,100}}
    ),
    graphics= {
      Rectangle(
        origin= {-100,2145.04},
        lineThickness= 1,
        pattern= LinePattern.None,
        fillPattern= FillPattern.Solid,
        fillColor= {230,230,230},
        extent= {{0.17,-2098.02},{200,-2245.04}},
        rotation= -0
      ),
      Line(
        origin= {-100,2145.04},
        color= {0,0,0},
        pattern= LinePattern.Solid,
        thickness= 0.25,
        points= {{193.86, -2181.04}, {198.41, -2166.55}, {196.24,
           -2148.38}},
        rotation= -0
```

```
    ),
    Line(
      origin= {-100,2145.04},
      color= {0,0,0},
      pattern= LinePattern.Solid,
      thickness= 0.25,
      points= {{189.41, -2181.04}, {184.86, -2166.55}, {187.89,
         -2147.95}},
      rotation= -0
    ),
    Ellipse(
      origin= {-100,2145.04},
      lineThickness= 0.86,
      pattern= LinePattern.None,
      fillPattern= FillPattern.Solid,
      fillColor= {219,0,0},
      extent= {{185.50,-2179.95},{198.35,-2192.80}},
      rotation= -0
    ),
    Line(
      origin= {-100,2145.04},
      color= {0,0,0},
      pattern= LinePattern.Solid,
      thickness= 0.25,
      points= {{193.86, -2109.77}, {198.41, -2124.27}, {196.24,
         -2142.44}},
      rotation= -0
    ),
    Line(
      origin= {-100,2145.04},
      color= {0,0,0},
      pattern= LinePattern.Solid,
      thickness= 0.25,
      points= {{189.41, -2109.77}, {184.86, -2124.27}, {187.89,
         -2142.87}},
      rotation= -0
    ),
    Line(
      origin= {-100,2145.04},
      color= {0,0,0},
      pattern= LinePattern.Solid,
      thickness= 0.25,
      points= {{10.57, -2181.04}, {15.12, -2166.55}, {12.95, -2148.38}},
      rotation= -0
    ),
    Line(
      origin= {-100,2145.04},
      color= {0,0,0},
      pattern= LinePattern.Solid,
      thickness= 0.25,
      points= {{6.12, -2181.04}, {1.58, -2166.55}, {4.60, -2147.95}},
      rotation= -0
    ),
    Ellipse(
      origin= {-100,2145.04},
      lineThickness= 0.86,
      pattern= LinePattern.None,
      fillPattern= FillPattern.Solid,
      fillColor= {219,0,0},
      extent= {{2.21,-2179.95},{15.06,-2192.80}},
      rotation= -0
    ),
```

```
  Line(
    origin= {-100,2145.04},
    color= {0,0,0},
    pattern= LinePattern.Solid,
    thickness= 0.25,
    points= {{10.57, -2109.77}, {15.12, -2124.27}, {12.95, -2142.44}},
    rotation= -0
  ),
  Line(
    origin= {-100,2145.04},
    color= {0,0,0},
    pattern= LinePattern.Solid,
    thickness= 0.25,
    points= {{6.12, -2109.77}, {1.58, -2124.27}, {4.60, -2142.87}},
    rotation= -0
  ),
  Line(
    origin= {-100,2145.04},
    color= {0,0,0},
    pattern= LinePattern.Solid,
    thickness= 0.25,
    points= {{100, -2045.04}, {100, -2098.03}},
    rotation= -0
  ),
  Line(
    origin= {-100,2145.04},
    color= {0,0,0},
    pattern= LinePattern.Solid,
    thickness= 0.25,
    points= {{25.85, -2181.04}, {30.39, -2166.55}, {28.23, -2148.38}},
    rotation= -0
  ),
  Line(
    origin= {-100,2145.04},
    color= {0,0,0},
    pattern= LinePattern.Solid,
    thickness= 0.25,
    points= {{21.39, -2181.04}, {16.85, -2166.55}, {19.88, -2147.95}},
    rotation= -0
  ),
  Ellipse(
    origin= {-100,2145.04},
    lineThickness= 0.86,
    pattern= LinePattern.None,
    fillPattern= FillPattern.Solid,
    fillColor= {219,0,0},
    extent= {{17.48,-2179.95},{30.33,-2192.80}},
    rotation= -0
  ),
  Line(
    origin= {-100,2145.04},
    color= {0,0,0},
    pattern= LinePattern.Solid,
    thickness= 0.25,
    points= {{25.85, -2109.77}, {30.39, -2124.27}, {28.23, -2142.44}},
    rotation= -0
  ),
  Line(
    origin= {-100,2145.04},
    color= {0,0,0},
    pattern= LinePattern.Solid,
    thickness= 0.25,
```

```
    points= {{21.39, -2109.77}, {16.85, -2124.27}, {19.88, -2142.87}},
    rotation= -0
  ),
  Line(
    origin= {-100,2145.04},
    color= {0,0,0},
    pattern= LinePattern.Solid,
    thickness= 0.25,
    points= {{41.12, -2181.04}, {45.67, -2166.55}, {43.50, -2148.38}},
    rotation= -0
  ),
  Line(
    origin= {-100,2145.04},
    color= {0,0,0},
    pattern= LinePattern.Solid,
    thickness= 0.25,
    points= {{36.67, -2181.04}, {32.12, -2166.55}, {35.15, -2147.95}},
    rotation= -0
  ),
  Ellipse(
    origin= {-100,2145.04},
    lineThickness= 0.86,
    pattern= LinePattern.None,
    fillPattern= FillPattern.Solid,
    fillColor= {219,0,0},
    extent= {{32.76,-2179.95},{45.60,-2192.80}},
    rotation= -0
  ),
  Line(
    origin= {-100,2145.04},
    color= {0,0,0},
    pattern= LinePattern.Solid,
    thickness= 0.25,
    points= {{41.12, -2109.77}, {45.67, -2124.27}, {43.50, -2142.44}},
    rotation= -0
  ),
  Line(
    origin= {-100,2145.04},
    color= {0,0,0},
    pattern= LinePattern.Solid,
    thickness= 0.25,
    points= {{36.67, -2109.77}, {32.12, -2124.27}, {35.15, -2142.87}},
    rotation= -0
  ),
  Line(
    origin= {-100,2145.04},
    color= {0,0,0},
    pattern= LinePattern.Solid,
    thickness= 0.25,
    points= {{56.40, -2181.04}, {60.94, -2166.55}, {58.78, -2148.38}},
    rotation= -0
  ),
  Line(
    origin= {-100,2145.04},
    color= {0,0,0},
    pattern= LinePattern.Solid,
    thickness= 0.25,
    points= {{51.94, -2181.04}, {47.40, -2166.55}, {50.43, -2147.95}},
    rotation= -0
  ),
  Ellipse(
    origin= {-100,2145.04},
```

```
    lineThickness= 0.86,
    pattern= LinePattern.None,
    fillPattern= FillPattern.Solid,
    fillColor= {219,0,0},
    extent= {{48.03,-2179.95},{60.88,-2192.80}},
    rotation= -0
  ),
  Line(
    origin= {-100,2145.04},
    color= {0,0,0},
    pattern= LinePattern.Solid,
    thickness= 0.25,
    points= {{56.40, -2109.77}, {60.94, -2124.27}, {58.78, -2142.44}},
    rotation= -0
  ),
  Line(
    origin= {-100,2145.04},
    color= {0,0,0},
    pattern= LinePattern.Solid,
    thickness= 0.25,
    points= {{51.94, -2109.77}, {47.40, -2124.27}, {50.43, -2142.87}},
    rotation= -0
  ),
  Line(
    origin= {-100,2145.04},
    color= {0,0,0},
    pattern= LinePattern.Solid,
    thickness= 0.25,
    points= {{71.67, -2181.04}, {76.21, -2166.55}, {74.05, -2148.38}},
    rotation= -0
  ),
  Line(
    origin= {-100,2145.04},
    color= {0,0,0},
    pattern= LinePattern.Solid,
    thickness= 0.25,
    points= {{67.21, -2181.04}, {62.67, -2166.55}, {65.70, -2147.95}},
    rotation= -0
  ),
  Ellipse(
    origin= {-100,2145.04},
    lineThickness= 0.86,
    pattern= LinePattern.None,
    fillPattern= FillPattern.Solid,
    fillColor= {219,0,0},
    extent= {{63.30,-2179.95},{76.15,-2192.80}},
    rotation= -0
  ),
  Line(
    origin= {-100,2145.04},
    color= {0,0,0},
    pattern= LinePattern.Solid,
    thickness= 0.25,
    points= {{71.67, -2109.77}, {76.21, -2124.27}, {74.05, -2142.44}},
    rotation= -0
  ),
  Line(
    origin= {-100,2145.04},
    color= {0,0,0},
    pattern= LinePattern.Solid,
    thickness= 0.25,
    points= {{67.21, -2109.77}, {62.67, -2124.27}, {65.70, -2142.87}},
```

```
    rotation= -0
  ),
  Line(
    origin= {-100,2145.04},
    color= {0,0,0},
    pattern= LinePattern.Solid,
    thickness= 0.25,
    points= {{86.95, -2181.04}, {91.49, -2166.55}, {89.32, -2148.38}},
    rotation= -0
  ),
  Line(
    origin= {-100,2145.04},
    color= {0,0,0},
    pattern= LinePattern.Solid,
    thickness= 0.25,
    points= {{82.49, -2181.04}, {77.95, -2166.55}, {80.97, -2147.95}},
    rotation= -0
  ),
  Ellipse(
    origin= {-100,2145.04},
    lineThickness= 0.86,
    pattern= LinePattern.None,
    fillPattern= FillPattern.Solid,
    fillColor= {219,0,0},
    extent= {{78.58,-2179.95},{91.43,-2192.80}},
    rotation= -0
  ),
  Line(
    origin= {-100,2145.04},
    color= {0,0,0},
    pattern= LinePattern.Solid,
    thickness= 0.25,
    points= {{86.95, -2109.77}, {91.49, -2124.27}, {89.32, -2142.44}},
    rotation= -0
  ),
  Line(
    origin= {-100,2145.04},
    color= {0,0,0},
    pattern= LinePattern.Solid,
    thickness= 0.25,
    points= {{82.49, -2109.77}, {77.95, -2124.27}, {80.97, -2142.87}},
    rotation= -0
  ),
  Line(
    origin= {-100,2145.04},
    color= {0,0,0},
    pattern= LinePattern.Solid,
    thickness= 0.25,
    points= {{102.22, -2181.04}, {106.76, -2166.55}, {104.60,
       -2148.38}},
    rotation= -0
  ),
  Line(
    origin= {-100,2145.04},
    color= {0,0,0},
    pattern= LinePattern.Solid,
    thickness= 0.25,
    points= {{97.76, -2181.04}, {93.22, -2166.55}, {96.25, -2147.95}},
    rotation= -0
  ),
  Ellipse(
    origin= {-100,2145.04},
```

```
    lineThickness= 0.86,
    pattern= LinePattern.None,
    fillPattern= FillPattern.Solid,
    fillColor= {219,0,0},
    extent= {{93.85,-2179.95},{106.70,-2192.80}},
    rotation= -0
  ),
  Line(
    origin= {-100,2145.04},
    color= {0,0,0},
    pattern= LinePattern.Solid,
    thickness= 0.25,
    points= {{102.22, -2109.77}, {106.76, -2124.27}, {104.60,
       -2142.44}},
    rotation= -0
  ),
  Line(
    origin= {-100,2145.04},
    color= {0,0,0},
    pattern= LinePattern.Solid,
    thickness= 0.25,
    points= {{97.76, -2109.77}, {93.22, -2124.27}, {96.25, -2142.87}},
    rotation= -0
  ),
  Line(
    origin= {-100,2145.04},
    color= {0,0,0},
    pattern= LinePattern.Solid,
    thickness= 0.25,
    points= {{117.49, -2181.04}, {122.04, -2166.55}, {119.87,
       -2148.38}},
    rotation= -0
  ),
  Line(
    origin= {-100,2145.04},
    color= {0,0,0},
    pattern= LinePattern.Solid,
    thickness= 0.25,
    points= {{113.04, -2181.04}, {108.49, -2166.55}, {111.52,
       -2147.95}},
    rotation= -0
  ),
  Ellipse(
    origin= {-100,2145.04},
    lineThickness= 0.86,
    pattern= LinePattern.None,
    fillPattern= FillPattern.Solid,
    fillColor= {219,0,0},
    extent= {{109.13,-2179.95},{121.98,-2192.80}},
    rotation= -0
  ),
  Line(
    origin= {-100,2145.04},
    color= {0,0,0},
    pattern= LinePattern.Solid,
    thickness= 0.25,
    points= {{117.49, -2109.77}, {122.04, -2124.27}, {119.87,
       -2142.44}},
    rotation= -0
  ),
  Line(
    origin= {-100,2145.04},
```

```
    color= {0,0,0},
    pattern= LinePattern.Solid,
    thickness= 0.25,
    points= {{113.04, -2109.77}, {108.49, -2124.27}, {111.52,
       -2142.87}},
    rotation= -0
  ),
  Line(
    origin= {-100,2145.04},
    color= {0,0,0},
    pattern= LinePattern.Solid,
    thickness= 0.25,
    points= {{132.77, -2181.04}, {137.31, -2166.55}, {135.15,
       -2148.38}},
    rotation= -0
  ),
  Line(
    origin= {-100,2145.04},
    color= {0,0,0},
    pattern= LinePattern.Solid,
    thickness= 0.25,
    points= {{128.31, -2181.04}, {123.77, -2166.55}, {126.80,
       -2147.95}},
    rotation= -0
  ),
  Ellipse(
    origin= {-100,2145.04},
    lineThickness= 0.86,
    pattern= LinePattern.None,
    fillPattern= FillPattern.Solid,
    fillColor= {219,0,0},
    extent= {{124.40,-2179.95},{137.25,-2192.80}},
    rotation= -0
  ),
  Line(
    origin= {-100,2145.04},
    color= {0,0,0},
    pattern= LinePattern.Solid,
    thickness= 0.25,
    points= {{132.77, -2109.77}, {137.31, -2124.27}, {135.15,
       -2142.44}},
    rotation= -0
  ),
  Line(
    origin= {-100,2145.04},
    color= {0,0,0},
    pattern= LinePattern.Solid,
    thickness= 0.25,
    points= {{128.31, -2109.77}, {123.77, -2124.27}, {126.80,
       -2142.87}},
    rotation= -0
  ),
  Line(
    origin= {-100,2145.04},
    color= {0,0,0},
    pattern= LinePattern.Solid,
    thickness= 0.25,
    points= {{148.04, -2181.04}, {152.58, -2166.55}, {150.42,
       -2148.38}},
    rotation= -0
  ),
  Line(
```

```
    origin= {-100,2145.04},
    color= {0,0,0},
    pattern= LinePattern.Solid,
    thickness= 0.25,
    points= {{143.59, -2181.04}, {139.04, -2166.55}, {142.07,
       -2147.95}},
    rotation= -0
  ),
  Ellipse(
    origin= {-100,2145.04},
    lineThickness= 0.86,
    pattern= LinePattern.None,
    fillPattern= FillPattern.Solid,
    fillColor= {219,0,0},
    extent= {{139.68,-2179.95},{152.52,-2192.80}},
    rotation= -0
  ),
  Line(
    origin= {-100,2145.04},
    color= {0,0,0},
    pattern= LinePattern.Solid,
    thickness= 0.25,
    points= {{148.04, -2109.77}, {152.58, -2124.27}, {150.42,
       -2142.44}},
    rotation= -0
  ),
  Line(
    origin= {-100,2145.04},
    color= {0,0,0},
    pattern= LinePattern.Solid,
    thickness= 0.25,
    points= {{143.59, -2109.77}, {139.04, -2124.27}, {142.07,
       -2142.87}},
    rotation= -0
  ),
  Line(
    origin= {-100,2145.04},
    color= {0,0,0},
    pattern= LinePattern.Solid,
    thickness= 0.25,
    points= {{163.32, -2181.04}, {167.86, -2166.55}, {165.69,
       -2148.38}},
    rotation= -0
  ),
  Line(
    origin= {-100,2145.04},
    color= {0,0,0},
    pattern= LinePattern.Solid,
    thickness= 0.25,
    points= {{158.86, -2181.04}, {154.32, -2166.55}, {157.35,
       -2147.95}},
    rotation= -0
  ),
  Ellipse(
    origin= {-100,2145.04},
    lineThickness= 0.86,
    pattern= LinePattern.None,
    fillPattern= FillPattern.Solid,
    fillColor= {219,0,0},
    extent= {{154.95,-2179.95},{167.80,-2192.80}},
    rotation= -0
  ),
```

```
Line(
  origin= {-100,2145.04},
  color= {0,0,0},
  pattern= LinePattern.Solid,
  thickness= 0.25,
  points= {{163.32, -2109.77}, {167.86, -2124.27}, {165.69,
     -2142.44}},
  rotation= -0
),
Line(
  origin= {-100,2145.04},
  color= {0,0,0},
  pattern= LinePattern.Solid,
  thickness= 0.25,
  points= {{158.86, -2109.77}, {154.32, -2124.27}, {157.35,
     -2142.87}},
  rotation= -0
),
Line(
  origin= {-100,2145.04},
  color= {0,0,0},
  pattern= LinePattern.Solid,
  thickness= 0.25,
  points= {{178.59, -2181.04}, {183.13, -2166.55}, {180.97,
     -2148.38}},
  rotation= -0
),
Line(
  origin= {-100,2145.04},
  color= {0,0,0},
  pattern= LinePattern.Solid,
  thickness= 0.25,
  points= {{174.13, -2181.04}, {169.59, -2166.55}, {172.62,
     -2147.95}},
  rotation= -0
),
Ellipse(
  origin= {-100,2145.04},
  lineThickness= 0.86,
  pattern= LinePattern.None,
  fillPattern= FillPattern.Solid,
  fillColor= {219,0,0},
  extent= {{170.22,-2179.95},{183.07,-2192.80}},
  rotation= -0
),
Line(
  origin= {-100,2145.04},
  color= {0,0,0},
  pattern= LinePattern.Solid,
  thickness= 0.25,
  points= {{178.59, -2109.77}, {183.13, -2124.27}, {180.97,
     -2142.44}},
  rotation= -0
),
Line(
  origin= {-100,2145.04},
  color= {0,0,0},
  pattern= LinePattern.Solid,
  thickness= 0.25,
  points= {{174.13, -2109.77}, {169.59, -2124.27}, {172.62,
     -2142.87}},
  rotation= -0
```

```
    ),
    Ellipse(
      origin= {-100,2145.04},
      lineThickness= 0.86,
      pattern= LinePattern.None,
      fillPattern= FillPattern.Solid,
      fillColor= {219,0,0},
      extent= {{185.50,-2098.02},{198.35,-2110.86}},
      rotation= -0
    ),
    Ellipse(
      origin= {-100,2145.04},
      lineThickness= 0.86,
      pattern= LinePattern.None,
      fillPattern= FillPattern.Solid,
      fillColor= {219,0,0},
      extent= {{2.21,-2098.02},{15.06,-2110.86}},
      rotation= -0
    ),
    Ellipse(
      origin= {-100,2145.04},
      lineThickness= 0.86,
      pattern= LinePattern.None,
      fillPattern= FillPattern.Solid,
      fillColor= {219,0,0},
      extent= {{17.48,-2098.02},{30.33,-2110.86}},
      rotation= -0
    ),
    Ellipse(
      origin= {-100,2145.04},
      lineThickness= 0.86,
      pattern= LinePattern.None,
      fillPattern= FillPattern.Solid,
      fillColor= {219,0,0},
      extent= {{32.76,-2098.02},{45.60,-2110.86}},
      rotation= -0
    ),
    Ellipse(
      origin= {-100,2145.04},
      lineThickness= 0.86,
      pattern= LinePattern.None,
      fillPattern= FillPattern.Solid,
      fillColor= {219,0,0},
      extent= {{48.03,-2098.02},{60.88,-2110.86}},
      rotation= -0
    ),
    Ellipse(
      origin= {-100,2145.04},
      lineThickness= 0.86,
      pattern= LinePattern.None,
      fillPattern= FillPattern.Solid,
      fillColor= {219,0,0},
      extent= {{63.30,-2098.02},{76.15,-2110.86}},
      rotation= -0
    ),
    Ellipse(
      origin= {-100,2145.04},
      lineThickness= 0.86,
      pattern= LinePattern.None,
      fillPattern= FillPattern.Solid,
      fillColor= {219,0,0},
      extent= {{78.58,-2098.02},{91.43,-2110.86}},
```

```
    rotation= -0
  ),
  Ellipse(
    origin= {-100,2145.04},
    lineThickness= 0.86,
    pattern= LinePattern.None,
    fillPattern= FillPattern.Solid,
    fillColor= {219,0,0},
    extent= {{93.85,-2098.02},{106.70,-2110.86}},
    rotation= -0
  ),
  Ellipse(
    origin= {-100,2145.04},
    lineThickness= 0.86,
    pattern= LinePattern.None,
    fillPattern= FillPattern.Solid,
    fillColor= {219,0,0},
    extent= {{109.13,-2098.02},{121.98,-2110.86}},
    rotation= -0
  ),
  Ellipse(
    origin= {-100,2145.04},
    lineThickness= 0.86,
    pattern= LinePattern.None,
    fillPattern= FillPattern.Solid,
    fillColor= {219,0,0},
    extent= {{124.40,-2098.02},{137.25,-2110.86}},
    rotation= -0
  ),
  Ellipse(
    origin= {-100,2145.04},
    lineThickness= 0.86,
    pattern= LinePattern.None,
    fillPattern= FillPattern.Solid,
    fillColor= {219,0,0},
    extent= {{139.68,-2098.02},{152.52,-2110.86}},
    rotation= -0
  ),
  Ellipse(
    origin= {-100,2145.04},
    lineThickness= 0.86,
    pattern= LinePattern.None,
    fillPattern= FillPattern.Solid,
    fillColor= {219,0,0},
    extent= {{154.95,-2098.02},{167.80,-2110.86}},
    rotation= -0
  ),
  Ellipse(
    origin= {-100,2145.04},
    lineThickness= 0.86,
    pattern= LinePattern.None,
    fillPattern= FillPattern.Solid,
    fillColor= {219,0,0},
    extent= {{170.22,-2098.02},{183.07,-2110.86}},
    rotation= -0
  ),
  Line(
    origin= {-100,2145.04},
    color= {0,0,0},
    pattern= LinePattern.Solid,
    thickness= 0.25,
    points= {{100, -2245.04}, {100, -2192.79}},
```

```
        rotation= -0
      )
    }
  )
);
end LipidBilayer;
```

Listing 10: `HHmodelica/Icons/OpenChannel.mo`

```
within HHmodelica.Icons;
model OpenChannel "pore that is open on both sides"
annotation(
  Icon(
    coordinateSystem(
      preserveAspectRatio= false,
      extent= {{-100,-100},{100,100}}
    ),
    graphics= {
      Polygon(
        origin= {-100,2145.04},
        lineThickness= 0.25,
        pattern= LinePattern.Solid,
        points= {{62.67, -2098.02}, {62.67, -2192.80}, {76.96, -2192.80},
            {82.99, -2186.78}, {82.99, -2145.04}, {82.99, -2115.02},
            {82.99, -2098.02}},
        fillPattern= FillPattern.Solid,
        fillColor= {124,154,239},
        lineColor= {0,0,0},
        rotation= -0
      ),
      Polygon(
        origin= {-100,2145.04},
        lineThickness= 0.25,
        pattern= LinePattern.Solid,
        points= {{137.43, -2098.02}, {137.43, -2192.80}, {123.13,
            -2192.80}, {117.11, -2186.78}, {117.11, -2145.04}, {117.11,
            -2115.02}, {117.11, -2098.02}},
        fillPattern= FillPattern.Solid,
        fillColor= {124,154,239},
        lineColor= {0,0,0},
        rotation= -0
      )
    }
  )
);
end OpenChannel;
```

Listing 11: `HHmodelica/Icons/Activatable.mo`

```
within HHmodelica.Icons;
model Activatable "pore that is open on the inside and closed on the
    outside"
annotation(
  Icon(
    coordinateSystem(
      preserveAspectRatio= false,
      extent= {{-100,-100},{100,100}}
    ),
    graphics= {
      Polygon(
        origin= {-100,2145.04},
        lineThickness= 0.25,
```

```
        pattern= LinePattern.Solid,
        points= {{62.67, -2098.02}, {62.67, -2192.80}, {76.96, -2192.80},
          {82.99, -2186.78}, {82.99, -2145.04}, {100.05, -2115.02},
          {100.05, -2098.02}},
        fillPattern= FillPattern.Solid,
        fillColor= {124,154,239},
        lineColor= {0,0,0},
        rotation= -0
      ),
      Polygon(
        origin= {-100,2145.04},
        lineThickness= 0.25,
        pattern= LinePattern.Solid,
        points= {{137.43, -2098.02}, {137.43, -2192.80}, {123.13,
          -2192.80}, {117.11, -2186.78}, {117.11, -2145.04}, {100.05,
          -2115.02}, {100.05, -2098.02}},
        fillPattern= FillPattern.Solid,
        fillColor= {124,154,239},
        lineColor= {0,0,0},
        rotation= -0
      )
    }
  )
);
end Activatable;
```

Listing 12: `HHmodelica/Icons/Inactivatable.mo`

```
within HHmodelica.Icons;
model Inactivatable "hinged lid for ion channel"
annotation(
  Icon(
    coordinateSystem(
      preserveAspectRatio= false,
      extent= {{-100,-100},{100,100}}
    ),
    graphics= {
      Polygon(
        origin= {-100,2145.04},
        lineThickness= 0.25,
        pattern= LinePattern.Solid,
        points= {{62.67, -2192.80}, {58.84, -2188.42}, {2.55, -2237.69},
          {6.38, -2242.07}, {17.17, -2232.62}, {25.67, -2233.19}, {51.34,
           -2210.71}, {51.91, -2202.22}},
        fillPattern= FillPattern.Solid,
        fillColor= {180,181,183},
        lineColor= {0,0,0},
        rotation= -0
      )
    }
  )
);
end Inactivatable;
```

Listing 13: `HHmodelica/Icons/IonChannel.mo`

```
within HHmodelica.Icons;
model IonChannel "base model for ion channel with gap in lipid bilayer"
annotation(
  Icon(
    coordinateSystem(
      preserveAspectRatio= false,
```

```
      extent= {{-100,-100},{100,100}}
    ),
    graphics= {
      Rectangle(
        origin= {-100,2145.04},
        lineThickness= 1,
        pattern= LinePattern.None,
        fillPattern= FillPattern.Solid,
        fillColor= {230,230,230},
        extent= {{0.22,-2098.02},{200.05,-2245.04}},
        rotation= -0
      ),
      Line(
        origin= {-100,2145.04},
        color= {0,0,0},
        pattern= LinePattern.Solid,
        thickness= 0.25,
        points= {{193.91, -2181.04}, {198.45, -2166.55}, {196.29,
          -2148.38}},
        rotation= -0
      ),
      Line(
        origin= {-100,2145.04},
        color= {0,0,0},
        pattern= LinePattern.Solid,
        thickness= 0.25,
        points= {{189.46, -2181.04}, {184.91, -2166.55}, {187.94,
          -2147.95}},
        rotation= -0
      ),
      Ellipse(
        origin= {-100,2145.04},
        lineThickness= 0.86,
        pattern= LinePattern.None,
        fillPattern= FillPattern.Solid,
        fillColor= {219,0,0},
        extent= {{185.55,-2179.95},{198.39,-2192.80}},
        rotation= -0
      ),
      Line(
        origin= {-100,2145.04},
        color= {0,0,0},
        pattern= LinePattern.Solid,
        thickness= 0.25,
        points= {{193.91, -2109.77}, {198.45, -2124.27}, {196.29,
          -2142.44}},
        rotation= -0
      ),
      Line(
        origin= {-100,2145.04},
        color= {0,0,0},
        pattern= LinePattern.Solid,
        thickness= 0.25,
        points= {{189.46, -2109.77}, {184.91, -2124.27}, {187.94,
          -2142.87}},
        rotation= -0
      ),
      Ellipse(
        origin= {-100,2145.04},
        lineThickness= 0.86,
        pattern= LinePattern.None,
        fillPattern= FillPattern.Solid,
```

```
    fillColor= {219,0,0},
    extent= {{185.55,-2098.02},{198.39,-2110.86}},
    rotation= -0
  ),
  Line(
    origin= {-100,2145.04},
    color= {0,0,0},
    pattern= LinePattern.Solid,
    thickness= 0.25,
    points= {{10.62, -2181.04}, {15.17, -2166.55}, {13, -2148.38}},
    rotation= -0
  ),
  Line(
    origin= {-100,2145.04},
    color= {0,0,0},
    pattern= LinePattern.Solid,
    thickness= 0.25,
    points= {{6.17, -2181.04}, {1.62, -2166.55}, {4.65, -2147.95}},
    rotation= -0
  ),
  Ellipse(
    origin= {-100,2145.04},
    lineThickness= 0.86,
    pattern= LinePattern.None,
    fillPattern= FillPattern.Solid,
    fillColor= {219,0,0},
    extent= {{2.26,-2179.95},{15.10,-2192.80}},
    rotation= -0
  ),
  Line(
    origin= {-100,2145.04},
    color= {0,0,0},
    pattern= LinePattern.Solid,
    thickness= 0.25,
    points= {{10.62, -2109.77}, {15.17, -2124.27}, {13, -2142.44}},
    rotation= -0
  ),
  Line(
    origin= {-100,2145.04},
    color= {0,0,0},
    pattern= LinePattern.Solid,
    thickness= 0.25,
    points= {{6.17, -2109.77}, {1.62, -2124.27}, {4.65, -2142.87}},
    rotation= -0
  ),
  Ellipse(
    origin= {-100,2145.04},
    lineThickness= 0.86,
    pattern= LinePattern.None,
    fillPattern= FillPattern.Solid,
    fillColor= {219,0,0},
    extent= {{2.26,-2098.02},{15.10,-2110.86}},
    rotation= -0
  ),
  Line(
    origin= {-100,2145.04},
    color= {0,0,0},
    pattern= LinePattern.Solid,
    thickness= 0.25,
    points= {{100.05, -2045.04}, {100.05, -2098.03}},
    rotation= -0
  ),
```

```
Line(
  origin= {-100,2145.04},
  color= {0,0,0},
  pattern= LinePattern.Solid,
  thickness= 0.25,
  points= {{25.90, -2181.04}, {30.44, -2166.55}, {28.28, -2148.38}},
  rotation= -0
),
Line(
  origin= {-100,2145.04},
  color= {0,0,0},
  pattern= LinePattern.Solid,
  thickness= 0.25,
  points= {{21.44, -2181.04}, {16.90, -2166.55}, {19.93, -2147.95}},
  rotation= -0
),
Ellipse(
  origin= {-100,2145.04},
  lineThickness= 0.86,
  pattern= LinePattern.None,
  fillPattern= FillPattern.Solid,
  fillColor= {219,0,0},
  extent= {{17.53,-2179.95},{30.38,-2192.80}},
  rotation= -0
),
Line(
  origin= {-100,2145.04},
  color= {0,0,0},
  pattern= LinePattern.Solid,
  thickness= 0.25,
  points= {{25.90, -2109.77}, {30.44, -2124.27}, {28.28, -2142.44}},
  rotation= -0
),
Line(
  origin= {-100,2145.04},
  color= {0,0,0},
  pattern= LinePattern.Solid,
  thickness= 0.25,
  points= {{21.44, -2109.77}, {16.90, -2124.27}, {19.93, -2142.87}},
  rotation= -0
),
Ellipse(
  origin= {-100,2145.04},
  lineThickness= 0.86,
  pattern= LinePattern.None,
  fillPattern= FillPattern.Solid,
  fillColor= {219,0,0},
  extent= {{17.53,-2098.02},{30.38,-2110.86}},
  rotation= -0
),
Line(
  origin= {-100,2145.04},
  color= {0,0,0},
  pattern= LinePattern.Solid,
  thickness= 0.25,
  points= {{41.17, -2181.04}, {45.71, -2166.55}, {43.55, -2148.38}},
  rotation= -0
),
Line(
  origin= {-100,2145.04},
  color= {0,0,0},
  pattern= LinePattern.Solid,
```

```
    thickness= 0.25,
    points= {{36.71, -2181.04}, {32.17, -2166.55}, {35.20, -2147.95}},
    rotation= -0
  ),
  Ellipse(
    origin= {-100,2145.04},
    lineThickness= 0.86,
    pattern= LinePattern.None,
    fillPattern= FillPattern.Solid,
    fillColor= {219,0,0},
    extent= {{32.80,-2179.95},{45.65,-2192.80}},
    rotation= -0
  ),
  Line(
    origin= {-100,2145.04},
    color= {0,0,0},
    pattern= LinePattern.Solid,
    thickness= 0.25,
    points= {{41.17, -2109.77}, {45.71, -2124.27}, {43.55, -2142.44}},
    rotation= -0
  ),
  Line(
    origin= {-100,2145.04},
    color= {0,0,0},
    pattern= LinePattern.Solid,
    thickness= 0.25,
    points= {{36.71, -2109.77}, {32.17, -2124.27}, {35.20, -2142.87}},
    rotation= -0
  ),
  Ellipse(
    origin= {-100,2145.04},
    lineThickness= 0.86,
    pattern= LinePattern.None,
    fillPattern= FillPattern.Solid,
    fillColor= {219,0,0},
    extent= {{32.80,-2098.02},{45.65,-2110.86}},
    rotation= -0
  ),
  Line(
    origin= {-100,2145.04},
    color= {0,0,0},
    pattern= LinePattern.Solid,
    thickness= 0.25,
    points= {{56.45, -2181.04}, {60.99, -2166.55}, {58.82, -2148.38}},
    rotation= -0
  ),
  Line(
    origin= {-100,2145.04},
    color= {0,0,0},
    pattern= LinePattern.Solid,
    thickness= 0.25,
    points= {{51.99, -2181.04}, {47.45, -2166.55}, {50.47, -2147.95}},
    rotation= -0
  ),
  Ellipse(
    origin= {-100,2145.04},
    lineThickness= 0.86,
    pattern= LinePattern.None,
    fillPattern= FillPattern.Solid,
    fillColor= {219,0,0},
    extent= {{48.08,-2179.95},{60.93,-2192.80}},
    rotation= -0
```

```
    ),
    Line(
      origin= {-100,2145.04},
      color= {0,0,0},
      pattern= LinePattern.Solid,
      thickness= 0.25,
      points= {{56.45, -2109.77}, {60.99, -2124.27}, {58.82, -2142.44}},
      rotation= -0
    ),
    Line(
      origin= {-100,2145.04},
      color= {0,0,0},
      pattern= LinePattern.Solid,
      thickness= 0.25,
      points= {{51.99, -2109.77}, {47.45, -2124.27}, {50.47, -2142.87}},
      rotation= -0
    ),
    Ellipse(
      origin= {-100,2145.04},
      lineThickness= 0.86,
      pattern= LinePattern.None,
      fillPattern= FillPattern.Solid,
      fillColor= {219,0,0},
      extent= {{48.08,-2098.02},{60.93,-2110.86}},
      rotation= -0
    ),
    Line(
      origin= {-100,2145.04},
      color= {0,0,0},
      pattern= LinePattern.Solid,
      thickness= 0.25,
      points= {{148.09, -2181.04}, {152.63, -2166.55}, {150.47,
        -2148.38}},
      rotation= -0
    ),
    Line(
      origin= {-100,2145.04},
      color= {0,0,0},
      pattern= LinePattern.Solid,
      thickness= 0.25,
      points= {{143.63, -2181.04}, {139.09, -2166.55}, {142.12,
        -2147.95}},
      rotation= -0
    ),
    Ellipse(
      origin= {-100,2145.04},
      lineThickness= 0.86,
      pattern= LinePattern.None,
      fillPattern= FillPattern.Solid,
      fillColor= {219,0,0},
      extent= {{139.72,-2179.95},{152.57,-2192.80}},
      rotation= -0
    ),
    Line(
      origin= {-100,2145.04},
      color= {0,0,0},
      pattern= LinePattern.Solid,
      thickness= 0.25,
      points= {{148.09, -2109.77}, {152.63, -2124.27}, {150.47,
        -2142.44}},
      rotation= -0
    ),
```

```
    Line(
      origin= {-100,2145.04},
      color= {0,0,0},
      pattern= LinePattern.Solid,
      thickness= 0.25,
      points= {{143.63, -2109.77}, {139.09, -2124.27}, {142.12,
         -2142.87}},
      rotation= -0
    ),
    Ellipse(
      origin= {-100,2145.04},
      lineThickness= 0.86,
      pattern= LinePattern.None,
      fillPattern= FillPattern.Solid,
      fillColor= {219,0,0},
      extent= {{139.72,-2098.02},{152.57,-2110.86}},
      rotation= -0
    ),
    Line(
      origin= {-100,2145.04},
      color= {0,0,0},
      pattern= LinePattern.Solid,
      thickness= 0.25,
      points= {{163.36, -2181.04}, {167.91, -2166.55}, {165.74,
         -2148.38}},
      rotation= -0
    ),
    Line(
      origin= {-100,2145.04},
      color= {0,0,0},
      pattern= LinePattern.Solid,
      thickness= 0.25,
      points= {{158.91, -2181.04}, {154.36, -2166.55}, {157.39,
         -2147.95}},
      rotation= -0
    ),
    Ellipse(
      origin= {-100,2145.04},
      lineThickness= 0.86,
      pattern= LinePattern.None,
      fillPattern= FillPattern.Solid,
      fillColor= {219,0,0},
      extent= {{155,-2179.95},{167.85,-2192.80}},
      rotation= -0
    ),
    Line(
      origin= {-100,2145.04},
      color= {0,0,0},
      pattern= LinePattern.Solid,
      thickness= 0.25,
      points= {{163.36, -2109.77}, {167.91, -2124.27}, {165.74,
         -2142.44}},
      rotation= -0
    ),
    Line(
      origin= {-100,2145.04},
      color= {0,0,0},
      pattern= LinePattern.Solid,
      thickness= 0.25,
      points= {{158.91, -2109.77}, {154.36, -2124.27}, {157.39,
         -2142.87}},
      rotation= -0
```

```
  ),
  Ellipse(
    origin= {-100,2145.04},
    lineThickness= 0.86,
    pattern= LinePattern.None,
    fillPattern= FillPattern.Solid,
    fillColor= {219,0,0},
    extent= {{155,-2098.02},{167.85,-2110.86}},
    rotation= -0
  ),
  Line(
    origin= {-100,2145.04},
    color= {0,0,0},
    pattern= LinePattern.Solid,
    thickness= 0.25,
    points= {{178.64, -2181.04}, {183.18, -2166.55}, {181.02,
      -2148.38}},
    rotation= -0
  ),
  Line(
    origin= {-100,2145.04},
    color= {0,0,0},
    pattern= LinePattern.Solid,
    thickness= 0.25,
    points= {{174.18, -2181.04}, {169.64, -2166.55}, {172.67,
      -2147.95}},
    rotation= -0
  ),
  Ellipse(
    origin= {-100,2145.04},
    lineThickness= 0.86,
    pattern= LinePattern.None,
    fillPattern= FillPattern.Solid,
    fillColor= {219,0,0},
    extent= {{170.27,-2179.95},{183.12,-2192.80}},
    rotation= -0
  ),
  Line(
    origin= {-100,2145.04},
    color= {0,0,0},
    pattern= LinePattern.Solid,
    thickness= 0.25,
    points= {{178.64, -2109.77}, {183.18, -2124.27}, {181.02,
      -2142.44}},
    rotation= -0
  ),
  Line(
    origin= {-100,2145.04},
    color= {0,0,0},
    pattern= LinePattern.Solid,
    thickness= 0.25,
    points= {{174.18, -2109.77}, {169.64, -2124.27}, {172.67,
      -2142.87}},
    rotation= -0
  ),
  Ellipse(
    origin= {-100,2145.04},
    lineThickness= 0.86,
    pattern= LinePattern.None,
    fillPattern= FillPattern.Solid,
    fillColor= {219,0,0},
    extent= {{170.27,-2098.02},{183.12,-2110.86}},
```

```
          rotation= -0
        ),
        Line(
          origin= {-100,2145.04},
          color= {0,0,0},
          pattern= LinePattern.Solid,
          thickness= 0.25,
          points= {{100.05, -2245.04}, {100.05, -2192.79}},
          rotation= -0
        )
      }
    )
  );
end IonChannel;
```

Listing 14: `HHmodelica/Icons/CurrentClamp.mo`

```
within HHmodelica.Icons;
model CurrentClamp "two electrodes sticking through lipid bilayer"
annotation(
  Icon(
    coordinateSystem(
      preserveAspectRatio= false,
      extent= {{-100,-100},{100,100}}
    ),
    graphics= {
      Line(
        origin= {-100,2145.04},
        color= {0,0,0},
        pattern= LinePattern.Solid,
        thickness= 0.25,
        points= {{132.77, -2181.04}, {137.31, -2166.55}, {135.15,
           -2148.38}},
        rotation= -0
      ),
      Rectangle(
        origin= {-100,2145.04},
        lineThickness= 1,
        pattern= LinePattern.None,
        fillPattern= FillPattern.Solid,
        fillColor= {230,230,230},
        extent= {{0.17,-2098.02},{200,-2245.04}},
        rotation= -0
      ),
      Line(
        origin= {-100,2145.04},
        color= {0,0,0},
        pattern= LinePattern.Solid,
        thickness= 0.25,
        points= {{193.86, -2181.04}, {198.41, -2166.55}, {196.24,
           -2148.38}},
        rotation= -0
      ),
      Line(
        origin= {-100,2145.04},
        color= {0,0,0},
        pattern= LinePattern.Solid,
        thickness= 0.25,
        points= {{189.41, -2181.04}, {184.86, -2166.55}, {187.89,
           -2147.95}},
        rotation= -0
      ),
```

```
Ellipse(
  origin= {-100,2145.04},
  lineThickness= 0.86,
  pattern= LinePattern.None,
  fillPattern= FillPattern.Solid,
  fillColor= {219,0,0},
  extent= {{185.50,-2179.95},{198.35,-2192.80}},
  rotation= -0
),
Line(
  origin= {-100,2145.04},
  color= {0,0,0},
  pattern= LinePattern.Solid,
  thickness= 0.25,
  points= {{10.57, -2181.04}, {15.12, -2166.55}, {12.95, -2148.38}},
  rotation= -0
),
Line(
  origin= {-100,2145.04},
  color= {0,0,0},
  pattern= LinePattern.Solid,
  thickness= 0.25,
  points= {{6.12, -2181.04}, {1.58, -2166.55}, {4.60, -2147.95}},
  rotation= -0
),
Ellipse(
  origin= {-100,2145.04},
  lineThickness= 0.86,
  pattern= LinePattern.None,
  fillPattern= FillPattern.Solid,
  fillColor= {219,0,0},
  extent= {{2.21,-2179.95},{15.06,-2192.80}},
  rotation= -0
),
Line(
  origin= {-100,2145.04},
  color= {0,0,0},
  pattern= LinePattern.Solid,
  thickness= 0.25,
  points= {{100, -2045.04}, {100, -2098.03}},
  rotation= -0
),
Line(
  origin= {-100,2145.04},
  color= {0,0,0},
  pattern= LinePattern.Solid,
  thickness= 0.25,
  points= {{25.85, -2181.04}, {30.39, -2166.55}, {28.23, -2148.38}},
  rotation= -0
),
Line(
  origin= {-100,2145.04},
  color= {0,0,0},
  pattern= LinePattern.Solid,
  thickness= 0.25,
  points= {{21.39, -2181.04}, {16.85, -2166.55}, {19.88, -2147.95}},
  rotation= -0
),
Ellipse(
  origin= {-100,2145.04},
  lineThickness= 0.86,
  pattern= LinePattern.None,
```

```
      fillPattern= FillPattern.Solid,
      fillColor= {219,0,0},
      extent= {{17.48,-2179.95},{30.33,-2192.80}},
      rotation= -0
    ),
    Line(
      origin= {-100,2145.04},
      color= {0,0,0},
      pattern= LinePattern.Solid,
      thickness= 0.25,
      points= {{41.12, -2181.04}, {45.67, -2166.55}, {43.50, -2148.38}},
      rotation= -0
    ),
    Line(
      origin= {-100,2145.04},
      color= {0,0,0},
      pattern= LinePattern.Solid,
      thickness= 0.25,
      points= {{36.67, -2181.04}, {32.12, -2166.55}, {35.15, -2147.95}},
      rotation= -0
    ),
    Ellipse(
      origin= {-100,2145.04},
      lineThickness= 0.86,
      pattern= LinePattern.None,
      fillPattern= FillPattern.Solid,
      fillColor= {219,0,0},
      extent= {{32.76,-2179.95},{45.60,-2192.80}},
      rotation= -0
    ),
    Line(
      origin= {-100,2145.04},
      color= {0,0,0},
      pattern= LinePattern.Solid,
      thickness= 0.25,
      points= {{56.40, -2181.04}, {60.94, -2166.55}, {58.78, -2148.38}},
      rotation= -0
    ),
    Line(
      origin= {-100,2145.04},
      color= {0,0,0},
      pattern= LinePattern.Solid,
      thickness= 0.25,
      points= {{51.94, -2181.04}, {47.40, -2166.55}, {50.43, -2147.95}},
      rotation= -0
    ),
    Ellipse(
      origin= {-100,2145.04},
      lineThickness= 0.86,
      pattern= LinePattern.None,
      fillPattern= FillPattern.Solid,
      fillColor= {219,0,0},
      extent= {{48.03,-2179.95},{60.88,-2192.80}},
      rotation= -0
    ),
    Line(
      origin= {-100,2145.04},
      color= {0,0,0},
      pattern= LinePattern.Solid,
      thickness= 0.25,
      points= {{71.67, -2181.04}, {76.21, -2166.55}, {74.05, -2148.38}},
      rotation= -0
```

```
),
Line(
  origin= {-100,2145.04},
  color= {0,0,0},
  pattern= LinePattern.Solid,
  thickness= 0.25,
  points= {{67.21, -2181.04}, {62.67, -2166.55}, {65.70, -2147.95}},
  rotation= -0
),
Ellipse(
  origin= {-100,2145.04},
  lineThickness= 0.86,
  pattern= LinePattern.None,
  fillPattern= FillPattern.Solid,
  fillColor= {219,0,0},
  extent= {{63.30,-2179.95},{76.15,-2192.80}},
  rotation= -0
),
Line(
  origin= {-100,2145.04},
  color= {0,0,0},
  pattern= LinePattern.Solid,
  thickness= 0.25,
  points= {{86.95, -2181.04}, {91.49, -2166.55}, {89.32, -2148.38}},
  rotation= -0
),
Line(
  origin= {-100,2145.04},
  color= {0,0,0},
  pattern= LinePattern.Solid,
  thickness= 0.25,
  points= {{82.49, -2181.04}, {77.95, -2166.55}, {80.97, -2147.95}},
  rotation= -0
),
Ellipse(
  origin= {-100,2145.04},
  lineThickness= 0.86,
  pattern= LinePattern.None,
  fillPattern= FillPattern.Solid,
  fillColor= {219,0,0},
  extent= {{78.58,-2179.95},{91.43,-2192.80}},
  rotation= -0
),
Line(
  origin= {-100,2145.04},
  color= {0,0,0},
  pattern= LinePattern.Solid,
  thickness= 0.25,
  points= {{102.22, -2181.04}, {106.76, -2166.55}, {104.60,
     -2148.38}},
  rotation= -0
),
Line(
  origin= {-100,2145.04},
  color= {0,0,0},
  pattern= LinePattern.Solid,
  thickness= 0.25,
  points= {{97.76, -2181.04}, {93.22, -2166.55}, {96.25, -2147.95}},
  rotation= -0
),
Ellipse(
  origin= {-100,2145.04},
```

```
          lineThickness= 0.86,
          pattern= LinePattern.None,
          fillPattern= FillPattern.Solid,
          fillColor= {219,0,0},
          extent= {{93.85,-2179.95},{106.70,-2192.80}},
          rotation= -0
        ),
        Line(
          origin= {-100,2145.04},
          color= {0,0,0},
          pattern= LinePattern.Solid,
          thickness= 0.25,
          points= {{117.49, -2181.04}, {122.04, -2166.55}, {119.87,
             -2148.38}},
          rotation= -0
        ),
        Line(
          origin= {-100,2145.04},
          color= {0,0,0},
          pattern= LinePattern.Solid,
          thickness= 0.25,
          points= {{113.04, -2181.04}, {108.49, -2166.55}, {111.52,
             -2147.95}},
          rotation= -0
        ),
        Ellipse(
          origin= {-100,2145.04},
          lineThickness= 0.86,
          pattern= LinePattern.None,
          fillPattern= FillPattern.Solid,
          fillColor= {219,0,0},
          extent= {{109.13,-2179.95},{121.98,-2192.80}},
          rotation= -0
        ),
        Line(
          origin= {-100,2145.04},
          color= {0,0,0},
          pattern= LinePattern.Solid,
          thickness= 0.25,
          points= {{128.31, -2181.04}, {123.77, -2166.55}, {126.80,
             -2147.95}},
          rotation= -0
        ),
        Ellipse(
          origin= {-100,2145.04},
          lineThickness= 0.86,
          pattern= LinePattern.None,
          fillPattern= FillPattern.Solid,
          fillColor= {219,0,0},
          extent= {{124.40,-2179.95},{137.25,-2192.80}},
          rotation= -0
        ),
        Line(
          origin= {-100,2145.04},
          color= {0,0,0},
          pattern= LinePattern.Solid,
          thickness= 0.25,
          points= {{148.04, -2181.04}, {152.58, -2166.55}, {150.42,
             -2148.38}},
          rotation= -0
        ),
        Line(
```

```
    origin= {-100,2145.04},
    color= {0,0,0},
    pattern= LinePattern.Solid,
    thickness= 0.25,
    points= {{143.59, -2181.04}, {139.04, -2166.55}, {142.07,
       -2147.95}},
    rotation= -0
  ),
  Ellipse(
    origin= {-100,2145.04},
    lineThickness= 0.86,
    pattern= LinePattern.None,
    fillPattern= FillPattern.Solid,
    fillColor= {219,0,0},
    extent= {{139.68,-2179.95},{152.52,-2192.80}},
    rotation= -0
  ),
  Line(
    origin= {-100,2145.04},
    color= {0,0,0},
    pattern= LinePattern.Solid,
    thickness= 0.25,
    points= {{163.32, -2181.04}, {167.86, -2166.55}, {165.69,
       -2148.38}},
    rotation= -0
  ),
  Line(
    origin= {-100,2145.04},
    color= {0,0,0},
    pattern= LinePattern.Solid,
    thickness= 0.25,
    points= {{158.86, -2181.04}, {154.32, -2166.55}, {157.35,
       -2147.95}},
    rotation= -0
  ),
  Ellipse(
    origin= {-100,2145.04},
    lineThickness= 0.86,
    pattern= LinePattern.None,
    fillPattern= FillPattern.Solid,
    fillColor= {219,0,0},
    extent= {{154.95,-2179.95},{167.80,-2192.80}},
    rotation= -0
  ),
  Line(
    origin= {-100,2145.04},
    color= {0,0,0},
    pattern= LinePattern.Solid,
    thickness= 0.25,
    points= {{178.59, -2181.04}, {183.13, -2166.55}, {180.97,
       -2148.38}},
    rotation= -0
  ),
  Line(
    origin= {-100,2145.04},
    color= {0,0,0},
    pattern= LinePattern.Solid,
    thickness= 0.25,
    points= {{174.13, -2181.04}, {169.59, -2166.55}, {172.62,
       -2147.95}},
    rotation= -0
  ),
```

```
Ellipse(
  origin= {-100,2145.04},
  lineThickness= 0.86,
  pattern= LinePattern.None,
  fillPattern= FillPattern.Solid,
  fillColor= {219,0,0},
  extent= {{170.22,-2179.95},{183.07,-2192.80}},
  rotation= -0
),
Line(
  origin= {-100,2145.04},
  color= {0,0,0},
  pattern= LinePattern.Solid,
  thickness= 0.25,
  points= {{100, -2245.04}, {100, -2192.79}},
  rotation= -0
),
Polygon(
  origin= {-100,2145.04},
  lineThickness= 0.25,
  pattern= LinePattern.Solid,
  points= {{198.69, -2060.29}, {187.99, -2085.82}, {113.84,
     -2218.63}, {154.72, -2071.87}, {165.42, -2046.35}},
  fillPattern= FillPattern.Solid,
  fillColor= {210,246,244},
  lineColor= {0,0,0},
  rotation= -0
),
Polygon(
  origin= {-100,2145.04},
  lineThickness= 0.25,
  pattern= LinePattern.Solid,
  points= {{1.31, -2060.29}, {12.01, -2085.82}, {86.16, -2218.63},
     {45.28, -2071.87}, {34.58, -2046.35}},
  fillPattern= FillPattern.Solid,
  fillColor= {210,246,244},
  lineColor= {0,0,0},
  rotation= -0
),
Line(
  origin= {-100,2145.04},
  color= {0,0,0},
  pattern= LinePattern.Solid,
  thickness= 0.25,
  points= {{193.86, -2109.77}, {198.41, -2124.27}, {196.24,
     -2142.44}},
  rotation= -0
),
Line(
  origin= {-100,2145.04},
  color= {0,0,0},
  pattern= LinePattern.Solid,
  thickness= 0.25,
  points= {{189.41, -2109.77}, {184.86, -2124.27}, {187.89,
     -2142.87}},
  rotation= -0
),
Ellipse(
  origin= {-100,2145.04},
  lineThickness= 0.86,
  pattern= LinePattern.None,
  fillPattern= FillPattern.Solid,
```

```
      fillColor= {219,0,0},
      extent= {{185.50,-2098.02},{198.35,-2110.86}},
      rotation= -0
    ),
    Line(
      origin= {-100,2145.04},
      color= {0,0,0},
      pattern= LinePattern.Solid,
      thickness= 0.25,
      points= {{10.57, -2109.77}, {15.12, -2124.27}, {12.95, -2142.44}},
      rotation= -0
    ),
    Line(
      origin= {-100,2145.04},
      color= {0,0,0},
      pattern= LinePattern.Solid,
      thickness= 0.25,
      points= {{6.12, -2109.77}, {1.58, -2124.27}, {4.60, -2142.87}},
      rotation= -0
    ),
    Ellipse(
      origin= {-100,2145.04},
      lineThickness= 0.86,
      pattern= LinePattern.None,
      fillPattern= FillPattern.Solid,
      fillColor= {219,0,0},
      extent= {{2.21,-2098.02},{15.06,-2110.86}},
      rotation= -0
    ),
    Line(
      origin= {-100,2145.04},
      color= {0,0,0},
      pattern= LinePattern.Solid,
      thickness= 0.25,
      points= {{25.85, -2109.77}, {30.39, -2124.27}, {28.23, -2142.44}},
      rotation= -0
    ),
    Line(
      origin= {-100,2145.04},
      color= {0,0,0},
      pattern= LinePattern.Solid,
      thickness= 0.25,
      points= {{21.39, -2109.77}, {16.85, -2124.27}, {19.88, -2142.87}},
      rotation= -0
    ),
    Ellipse(
      origin= {-100,2145.04},
      lineThickness= 0.86,
      pattern= LinePattern.None,
      fillPattern= FillPattern.Solid,
      fillColor= {219,0,0},
      extent= {{17.48,-2098.02},{30.33,-2110.86}},
      rotation= -0
    ),
    Line(
      origin= {-100,2145.04},
      color= {0,0,0},
      pattern= LinePattern.Solid,
      thickness= 0.25,
      points= {{41.12, -2109.77}, {45.67, -2124.27}, {43.50, -2142.44}},
      rotation= -0
    ),
```

```
Line(
  origin= {-100,2145.04},
  color= {0,0,0},
  pattern= LinePattern.Solid,
  thickness= 0.25,
  points= {{36.67, -2109.77}, {32.12, -2124.27}, {35.15, -2142.87}},
  rotation= -0
),
Ellipse(
  origin= {-100,2145.04},
  lineThickness= 0.86,
  pattern= LinePattern.None,
  fillPattern= FillPattern.Solid,
  fillColor= {219,0,0},
  extent= {{32.76,-2098.02},{45.60,-2110.86}},
  rotation= -0
),
Line(
  origin= {-100,2145.04},
  color= {0,0,0},
  pattern= LinePattern.Solid,
  thickness= 0.25,
  points= {{56.40, -2109.77}, {60.94, -2124.27}, {58.78, -2142.44}},
  rotation= -0
),
Line(
  origin= {-100,2145.04},
  color= {0,0,0},
  pattern= LinePattern.Solid,
  thickness= 0.25,
  points= {{51.94, -2109.77}, {47.40, -2124.27}, {50.43, -2142.87}},
  rotation= -0
),
Ellipse(
  origin= {-100,2145.04},
  lineThickness= 0.86,
  pattern= LinePattern.None,
  fillPattern= FillPattern.Solid,
  fillColor= {219,0,0},
  extent= {{48.03,-2098.02},{60.88,-2110.86}},
  rotation= -0
),
Line(
  origin= {-100,2145.04},
  color= {0,0,0},
  pattern= LinePattern.Solid,
  thickness= 0.25,
  points= {{71.67, -2109.77}, {76.21, -2124.27}, {74.05, -2142.44}},
  rotation= -0
),
Line(
  origin= {-100,2145.04},
  color= {0,0,0},
  pattern= LinePattern.Solid,
  thickness= 0.25,
  points= {{67.21, -2109.77}, {62.67, -2124.27}, {65.70, -2142.87}},
  rotation= -0
),
Ellipse(
  origin= {-100,2145.04},
  lineThickness= 0.86,
  pattern= LinePattern.None,
```

```
      fillPattern= FillPattern.Solid,
      fillColor= {219,0,0},
      extent= {{63.30,-2098.02},{76.15,-2110.86}},
      rotation= -0
    ),
    Line(
      origin= {-100,2145.04},
      color= {0,0,0},
      pattern= LinePattern.Solid,
      thickness= 0.25,
      points= {{86.95, -2109.77}, {91.49, -2124.27}, {89.32, -2142.44}},
      rotation= -0
    ),
    Line(
      origin= {-100,2145.04},
      color= {0,0,0},
      pattern= LinePattern.Solid,
      thickness= 0.25,
      points= {{82.49, -2109.77}, {77.95, -2124.27}, {80.97, -2142.87}},
      rotation= -0
    ),
    Ellipse(
      origin= {-100,2145.04},
      lineThickness= 0.86,
      pattern= LinePattern.None,
      fillPattern= FillPattern.Solid,
      fillColor= {219,0,0},
      extent= {{78.58,-2098.02},{91.43,-2110.86}},
      rotation= -0
    ),
    Line(
      origin= {-100,2145.04},
      color= {0,0,0},
      pattern= LinePattern.Solid,
      thickness= 0.25,
      points= {{102.22, -2109.77}, {106.76, -2124.27}, {104.60,
        -2142.44}},
      rotation= -0
    ),
    Line(
      origin= {-100,2145.04},
      color= {0,0,0},
      pattern= LinePattern.Solid,
      thickness= 0.25,
      points= {{97.76, -2109.77}, {93.22, -2124.27}, {96.25, -2142.87}},
      rotation= -0
    ),
    Ellipse(
      origin= {-100,2145.04},
      lineThickness= 0.86,
      pattern= LinePattern.None,
      fillPattern= FillPattern.Solid,
      fillColor= {219,0,0},
      extent= {{93.85,-2098.02},{106.70,-2110.86}},
      rotation= -0
    ),
    Line(
      origin= {-100,2145.04},
      color= {0,0,0},
      pattern= LinePattern.Solid,
      thickness= 0.25,
```

```
    points= {{117.49, -2109.77}, {122.04, -2124.27}, {119.87,
        -2142.44}},
    rotation= -0
  ),
  Line(
    origin= {-100,2145.04},
    color= {0,0,0},
    pattern= LinePattern.Solid,
    thickness= 0.25,
    points= {{113.04, -2109.77}, {108.49, -2124.27}, {111.52,
        -2142.87}},
    rotation= -0
  ),
  Ellipse(
    origin= {-100,2145.04},
    lineThickness= 0.86,
    pattern= LinePattern.None,
    fillPattern= FillPattern.Solid,
    fillColor= {219,0,0},
    extent= {{109.13,-2098.02},{121.98,-2110.86}},
    rotation= -0
  ),
  Line(
    origin= {-100,2145.04},
    color= {0,0,0},
    pattern= LinePattern.Solid,
    thickness= 0.25,
    points= {{132.77, -2109.77}, {137.31, -2124.27}, {135.15,
        -2142.44}},
    rotation= -0
  ),
  Line(
    origin= {-100,2145.04},
    color= {0,0,0},
    pattern= LinePattern.Solid,
    thickness= 0.25,
    points= {{128.31, -2109.77}, {123.77, -2124.27}, {126.80,
        -2142.87}},
    rotation= -0
  ),
  Ellipse(
    origin= {-100,2145.04},
    lineThickness= 0.86,
    pattern= LinePattern.None,
    fillPattern= FillPattern.Solid,
    fillColor= {219,0,0},
    extent= {{124.40,-2098.02},{137.25,-2110.86}},
    rotation= -0
  ),
  Line(
    origin= {-100,2145.04},
    color= {0,0,0},
    pattern= LinePattern.Solid,
    thickness= 0.25,
    points= {{148.04, -2109.77}, {152.58, -2124.27}, {150.42,
        -2142.44}},
    rotation= -0
  ),
  Line(
    origin= {-100,2145.04},
    color= {0,0,0},
    pattern= LinePattern.Solid,
```

```
    thickness= 0.25,
    points= {{143.59, -2109.77}, {139.04, -2124.27}, {142.07,
       -2142.87}},
    rotation= -0
  ),
  Ellipse(
    origin= {-100,2145.04},
    lineThickness= 0.86,
    pattern= LinePattern.None,
    fillPattern= FillPattern.Solid,
    fillColor= {219,0,0},
    extent= {{139.68,-2098.02},{152.52,-2110.86}},
    rotation= -0
  ),
  Line(
    origin= {-100,2145.04},
    color= {0,0,0},
    pattern= LinePattern.Solid,
    thickness= 0.25,
    points= {{163.32, -2109.77}, {167.86, -2124.27}, {165.69,
       -2142.44}},
    rotation= -0
  ),
  Line(
    origin= {-100,2145.04},
    color= {0,0,0},
    pattern= LinePattern.Solid,
    thickness= 0.25,
    points= {{158.86, -2109.77}, {154.32, -2124.27}, {157.35,
       -2142.87}},
    rotation= -0
  ),
  Ellipse(
    origin= {-100,2145.04},
    lineThickness= 0.86,
    pattern= LinePattern.None,
    fillPattern= FillPattern.Solid,
    fillColor= {219,0,0},
    extent= {{154.95,-2098.02},{167.80,-2110.86}},
    rotation= -0
  ),
  Line(
    origin= {-100,2145.04},
    color= {0,0,0},
    pattern= LinePattern.Solid,
    thickness= 0.25,
    points= {{178.59, -2109.77}, {183.13, -2124.27}, {180.97,
       -2142.44}},
    rotation= -0
  ),
  Line(
    origin= {-100,2145.04},
    color= {0,0,0},
    pattern= LinePattern.Solid,
    thickness= 0.25,
    points= {{174.13, -2109.77}, {169.59, -2124.27}, {172.62,
       -2142.87}},
    rotation= -0
  ),
  Ellipse(
    origin= {-100,2145.04},
    lineThickness= 0.86,
```

```
        pattern= LinePattern.None,
        fillPattern= FillPattern.Solid,
        fillColor= {219,0,0},
        extent= {{170.22,-2098.02},{183.07,-2110.86}},
        rotation= -0
      )
    }
  )
);
end CurrentClamp;
```
